# Graphene‐Based Liquid Cell Designs for In Situ Liquid‐Phase Transmission Electron Microscopy: Recent Developments and Perspectives

**DOI:** 10.1002/smsc.202500333

**Published:** 2025-09-22

**Authors:** Hyeonjong Ma, Hyeongseung Kim, Jiwoong Yang

**Affiliations:** ^1^ Department of Energy Science and Engineering Daegu Gyeongbuk Institute of Science and Technology (DGIST) Daegu 42988 Republic of Korea; ^2^ Materials Sciences Division Lawrence Berkeley National Laboratory Berkeley 94720 CA USA; ^3^ Energy Science and Engineering Research Center Daegu Gyeongbuk Institute of Science and Technology (DGIST) Daegu 42988 Republic of Korea

**Keywords:** cell design, graphene liquid cells, in situ transmission electron microscopy, liquid‐phase transmission electron microscopy, nanoscale dynamics

## Abstract

Recent advances in liquid‐phase transmission electron microscopy (TEM) have enabled the direct visualization of reaction pathways of nanomaterials, providing critical insights into diverse nanoscale processes such as crystallization, phase transition, shape transformation, etching, and nanoparticle motions. Among various liquid cells, graphene liquid cells (GLCs) are particularly advantageous due to the intrinsic properties of graphene—high electrical and thermal conductivity, exceptional mechanical flexibility, and radical scavenging effects—which allow atomic‐scale spatial resolution and enhanced imaging stability. This review article highlights the recent progress in GLC‐based liquid‐phase TEM, focusing on the evolution of structural designs, including veil‐type, well‐type, liquid‐flowing‐type, and mixing‐type GLCs. Each configuration offers unique advantages tailored to observing distinct types of nanoscale dynamic processes. These studies have elucidated both classical reaction pathways and complex, nonclassical mechanisms involving transient intermediates. Overall, this review highlights how developments in GLC designs have significantly advanced the capabilities of in situ liquid‐phase TEM, providing unprecedented opportunities to study nanoscale processes at atomic resolution.

## Graphical abstract

This review highlights the advances in graphene liquid cell designs for in situ liquid‐phase transmission electron microscopy, spanning veil‐type, well‐type, liquid‐flowing‐type, and mixing‐type configurations. These developments collectively enable real‐time visualization of diverse reactions in liquid environments at atomic resolution, thereby providing valuable insights into a wide range of liquid‐phase nanoscale processes.

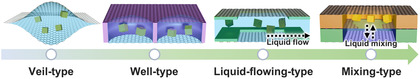

## 1 Introduction

Understanding nanoscale dynamic processes, such as nucleation, growth, phase transformations, and deformations of nanomaterials, is fundamentally important across various disciplines, including materials science,^[^ ^1^, ^2^, ^3^, ^4^, ^5^ ^]^ chemistry,^[^ ^6^, ^7^ ^]^ physics,^[^ ^8^, ^9^ ^]^ and nanotechnology.^[^ ^10^, ^11^, ^12^, ^13^, ^14^, ^15^, ^16^ ^]^ Traditional analytical techniques, such as optical spectroscopy,^[^ ^17^, ^18^, ^19^, ^20^, ^21^ ^]^ X‐ray‐based methods,^[^ ^22^, ^23^, ^24^, ^25^, ^26^, ^27^ ^]^ and electron microscopy‐based approaches^[^ ^28^, ^29^, ^30^ ^]^ (e.g., TEM, SEM, and AFM), typically provide ensemble‐averaged information or ex situ characterization of structural changes, but often fail to capture critical localized transformations occurring within individual nanoparticles, such as the formation of transient defects, intermediate species, or subtle structural rearrangements essential for fully understanding reaction pathways. In contrast, in situ TEM enables direct and real‐time visualization of dynamic processes with high spatial resolution, providing detailed insights into structural evolution at the atomic scale. Moreover, this method facilitates the tracking of individual nanoparticles or interfaces, thereby revealing transient reaction intermediates or transition states that are otherwise difficult to detect.

Conventional TEM setups require high‐vacuum conditions, limiting observations primarily to reactions occurring in solid‐state materials^[^ ^31^, ^32^, ^33^, ^34^, ^35^, ^36^, ^37^, ^38^, ^39^, ^40^ ^]^ or involving nonvolatile liquids that do not evaporate under vacuum conditions.^[^ ^41^, ^42^, ^43^ ^]^ Recent advances in closed‐cell systems, which isolate liquid‐ or gas‐phase samples from the vacuum environment, have significantly expanded the range of reactions accessible to in situ TEM studies.^[^ ^44^, ^45^, ^46^ ^]^ For example, microfabricated liquid cells equipped with silicon nitride (SiN_*x*_) windows have become widely used for in situ liquid‐phase TEM studies of various nanoscale processes, including nanocrystal crystallization,^[^ ^47^, ^48^, ^49^, ^50^, ^51^, ^52^, ^53^, ^54^, ^55^, ^56^, ^57^, ^58^, ^59^, ^60^, ^61^, ^62^, ^63^, ^64^ ^]^ motions,^[^ ^65^, ^66^, ^67^, ^68^, ^69^ ^]^ etching,^[^ ^70^, ^71^, ^72^, ^73^ ^]^ and structural transformations.^[^ ^74^, ^75^, ^76^, ^77^, ^78^, ^79^ ^]^ These cells also facilitate the controlled introduction of reactants during imaging, allowing precise manipulation of reaction conditions. Furthermore, microfabricated liquid cells have also been developed by integrating electrodes^[^ ^80^ ^]^ to enable investigation of electrochemical reactions in fuel cells,^[^ ^81^ ^]^ electrolyzers,^[^ ^82^, ^83^, ^84^ ^]^ and batteries.^[^ ^85^, ^86^, ^87^ ^]^ However, window membranes such as SiN_*x*_ are typically tens of nanometers thick, causing significant electron scattering and consequently reducing imaging resolution and contrast, particularly for low atomic number (low‐Z) materials.^[^ ^60^, ^61^ ^]^ Moreover, SiN_*x*_ membranes exhibit an inherent brittleness,^[^ ^88^, ^89^ ^]^ which makes them susceptible to fracture during liquid cell fabrication and TEM imaging. Additionally, liquid flow in these systems often induces sample drift, and their single‐chamber design restricts experimental flexibility, as all reactions must proceed simultaneously across the entire sample during a single experiment.

**Figure 1:**
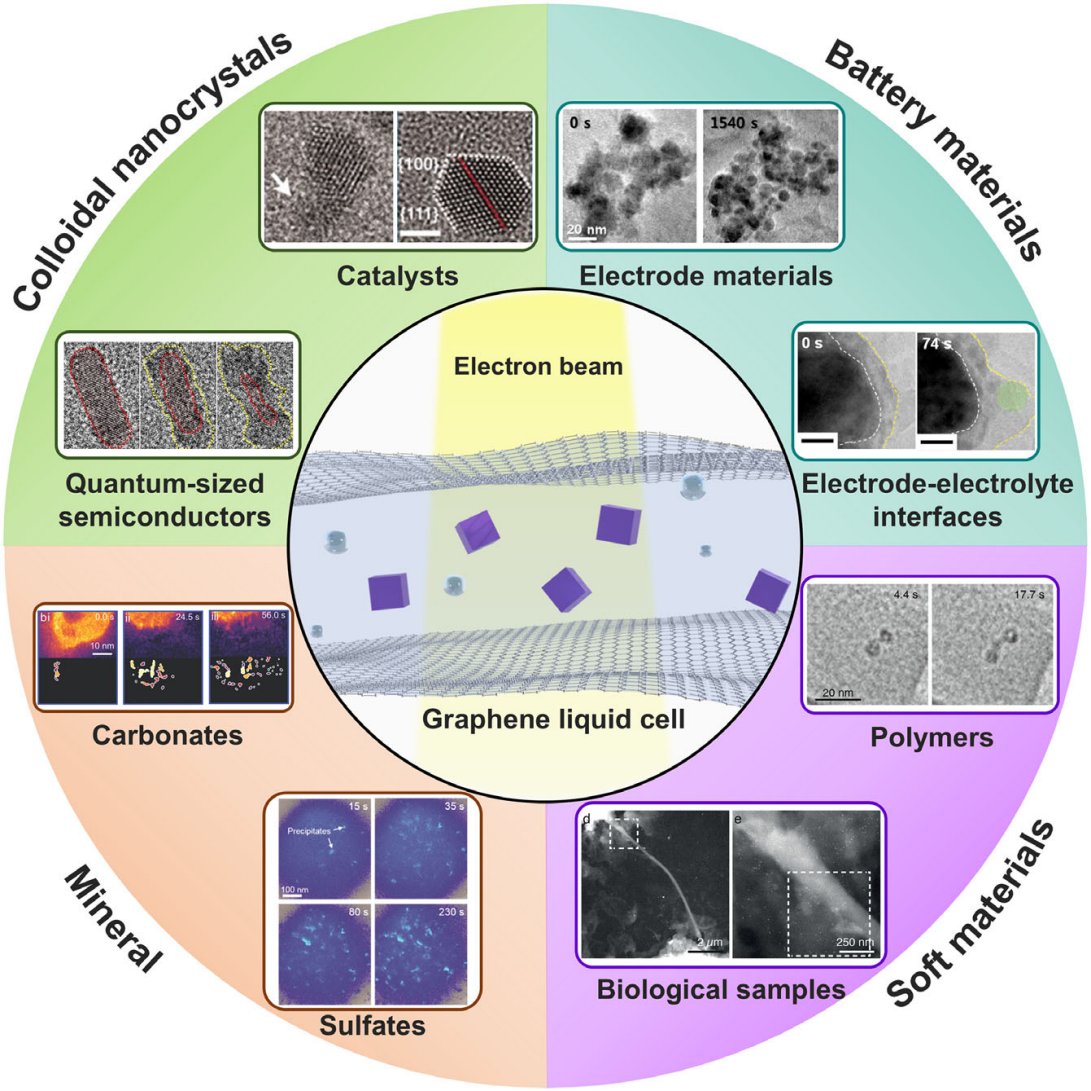
Schematic presentation of representative applications of in situ GLC‐TEM. Catalysts: Reproduced with permission.^[^ ^91^ ^]^ Copyright 2012, AAAS. Quantum‐sized semiconductors: Reproduced with permission.^[^ ^103^ ^]^ Copyright 2023, American Chemical Society. Electrode materials: Reproduced with permission.^[^ ^108^ ^]^ Copyright 2017, American Chemical Society. Electrode‐electrolyte interfaces: Reproduced with permission.^[^ ^110^ ^]^ Copyright 2016, Elsevier. Carbonates: Reproduced with permission.^[^ ^111^ ^]^ Copyright 2021, Wiley‐VCH. Sulfates: Reproduced with permission.^[^ ^112^ ^]^ Copyright 2016, American Chemical Society. Polymers: Reproduced with permission.^[^ ^114^ ^]^ Copyright 2022, Wiley‐VCH. Biological samples: Reproduced with permission.^[^ ^116^ ^]^ Copyright 2017, American Chemical Society.

**Figure 2:**
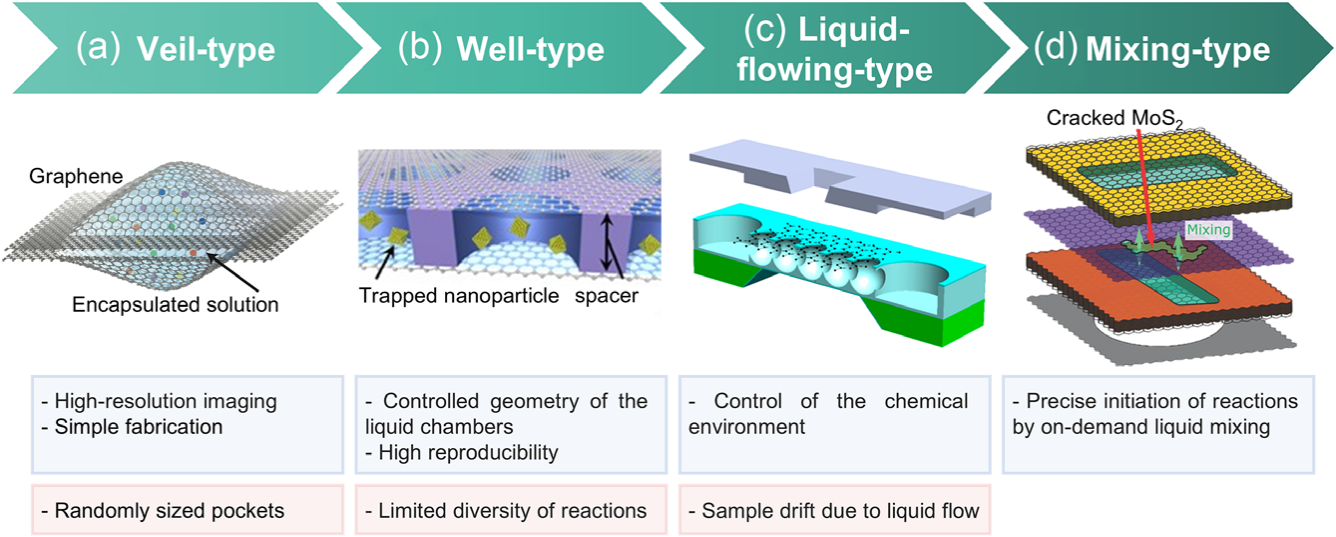
Schematic overview illustrating the evolution of GLC structures. a) Veil‐type GLCs. Reproduced with permission.^[^ ^91^ ^]^ Copyright 2012, AAAS. b) Well‐type GLCs. Reproduced with permission.^[^ ^167^ ^]^ Copyright 2016, Wiley‐VCH. c) Liquid‐flowing‐type GLCs. Reproduced with permission.^[^ ^191^ ^]^ Copyright 2020, American Chemical Society. d) Mixing‐type GLCs. Reproduced with permission.^[^ ^111^ ^]^ Copyright 2021, Wiley‐VCH. Advantages and limitations for each GLC configuration are summarized below the respective schematics.

To address these challenges, atomically thin 2D membranes, particularly graphene, have emerged as alternative window materials for liquid cells. Specifically, graphene liquid cells (GLCs) offer exceptional electron transparency,^[^ ^90^ ^]^ reduced background scattering, and simplified fabrication methods, enabling high‐resolution imaging of nanoscale reaction trajectories with improved contrast. Accordingly, advancements in GLC technology have significantly enhanced our understanding of liquid‐phase dynamic processes involving colloidal nanocrystals,^[^ ^91^, ^92^, ^93^, ^94^, ^95^, ^96^, ^97^, ^98^, ^99^, ^100^, ^101^, ^102^, ^103^, ^104^ ^]^ battery materials,^[^ ^105^, ^106^, ^107^, ^108^, ^109^, ^110^ ^]^ minerals,^[^ ^111^, ^112^, ^113^ ^]^ and soft materials^[^ ^114^, ^115^, ^116^, ^117^, ^118^, ^119^ ^]^ (**Figure** **1**). This review article highlights recent progress in GLC TEM technologies, emphasizing the systematic structural evolution and optimization of GLCs, including i) veil‐type, ii) well‐type, iii) liquid‐flowing‐type, and iv) mixing‐type configurations (**Figure** **2**). These advancements have considerably improved our ability to study nanoscale processes in liquid‐phase environments, thereby opening new opportunities to investigate previously inaccessible reaction mechanisms. By enabling the direct observation of complex liquid‐phase processes at atomic resolution, graphene‐based liquid cells provide deeper insights into fundamental nanoscale phenomena.

## 2 Veil‐Type GLCs

**Figure 3:**
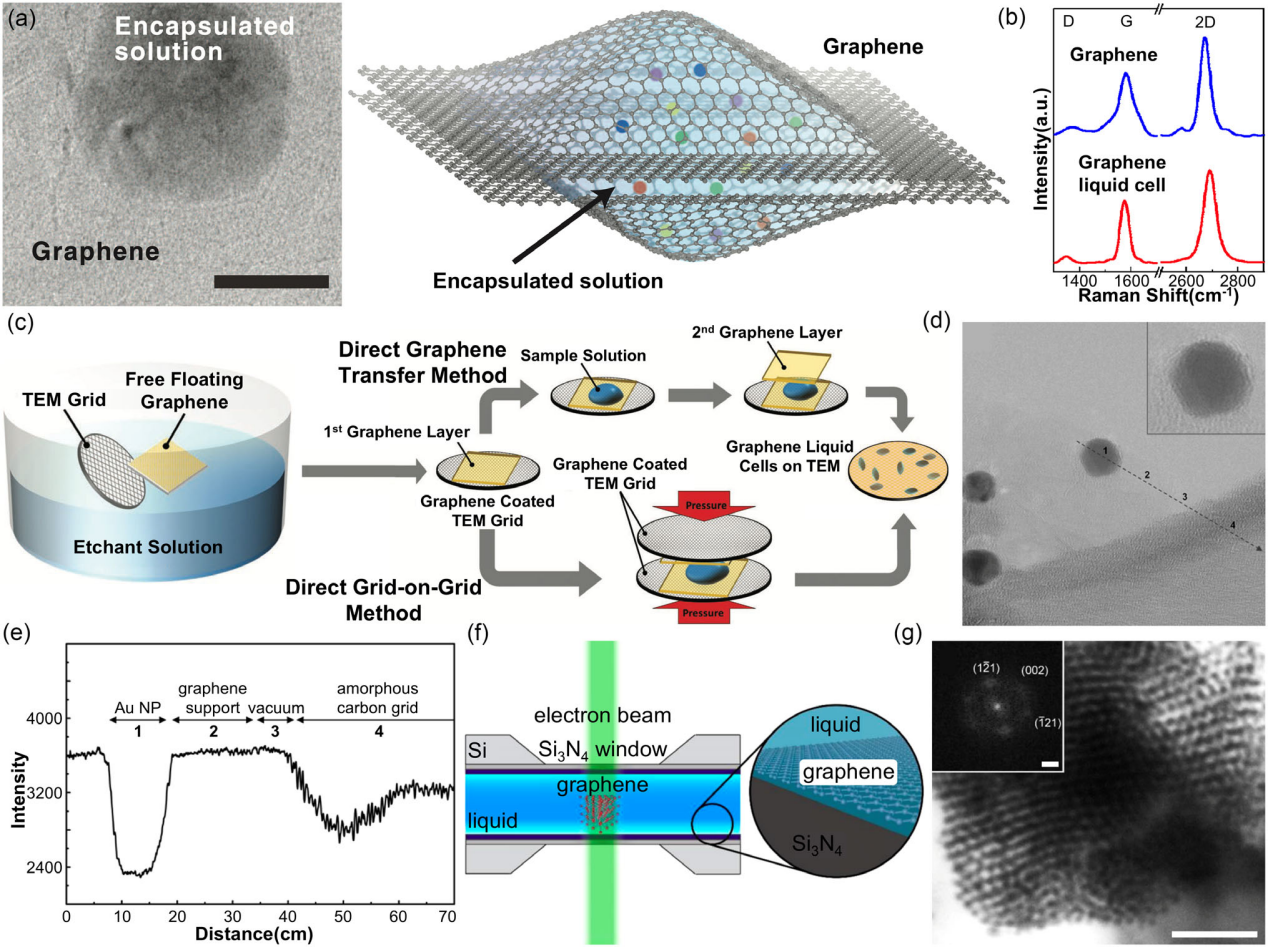
Structure and fabrication of veil‐type GLCs. a) TEM image showing two stacked graphene sheets enclosing a liquid blister and schematic illustration of GLCs. Scale bar: 50 nm. b) Raman spectra of graphene on a TEM grid and within GLCs. Reproduced with permission.^[^ ^91^ ^]^ Copyright 2012, AAAS. c) Fabrication procedure for veil‐type GLCs. Reproduced with permission.^[^ ^47^ ^]^ Copyright 2019, Wiley‐VCH. d) High‐magnification TEM image of citrate‐capped Au nanoparticles, showing the edge of a graphene sheet (arrow). e) Intensity line profile from (d) along the arrow. Reproduced with permission.^[^ ^90^ ^]^ Copyright 2009, American Chemical Society. f) Schematic illustrating graphene‐coated Si_3_N_4_ liquid cells. g) Low‐magnification TEM image of DNA‐AuNP superlattices. Scale bar: 100 nm. Reproduced with permission.^[^ ^125^ ^]^ Copyright 2017, American Chemical Society.

Encapsulating liquid‐phase solutions and isolating them from the high‐vacuum environment of the TEM column using thin, electron‐transparent membranes is essential for liquid‐phase TEM imaging. Among various membranes, graphene is particularly advantageous for liquid‐phase TEM imaging due to its distinctive physical and electronic properties. Graphene membranes, composed of carbon atoms (*Z* = 6) arranged in a honeycomb lattice, exhibit exceptional liquid impermeability and robust interlayer adhesion through van der Waals forces,^[^ ^120^ ^]^ effectively encapsulating liquid samples and preventing their evaporation.^[^ ^121^ ^]^ Yuk et al. introduced veil‐type GLCs by encapsulating liquid solutions between two graphene membranes.^[^ ^91^ ^]^ **Figure** **3**a schematically illustrates the configuration of veil‐type GLCs, in which liquid samples are sealed between two graphene membranes. Graphene can bend and conform to liquid droplets,^[^ ^122^ ^]^ enabling their effective encapsulation.

Veil‐type GLCs are fabricated using a direct transfer method.^[^ ^123^ ^]^ Typically, graphene synthesized by chemical vapor deposition (CVD) on a Cu substrate is separated using an aqueous FeCl_3_ or ammonium persulfate etching solution. Subsequently, floating graphene sheets are thoroughly rinsed several times with deionized water to remove Cu residues and contaminants before being transferred onto a TEM grid. This direct transfer method effectively minimizes damage to the graphene sheets (Figure 3b). Subsequent fabrication involves depositing liquid droplets onto a graphene‐coated TEM grid, followed by encapsulation with an additional freestanding graphene sheet. Alternatively, two graphene‐coated TEM grids are assembled with liquid droplets sandwiched between them, forming a stable, sealed structure maintained by van der Waals interactions (Figure 3c).^[^ ^45^, ^124^ ^]^ This design accommodates a wide range of liquid thicknesses, typically on the order of tens of nanometers.

Because veil‐type GLCs use atomically thin graphene membranes as imaging windows and liquid‐sealing membranes, they offer distinct advantages over microfabricated SiN_*x*_ liquid cells. First, the high electron transparency of graphene significantly enhances imaging contrast while effectively minimizing electron scattering compared to SiN_*x*_ membranes and amorphous carbon films.^[^ ^90^ ^]^ Consequently, graphene sheets are nearly indistinguishable from regions without any supporting membrane (Figure 3d,e). Second, graphene can effectively scavenge radiolysis products generated by electron beam irradiation of the enclosed liquid medium (Figure 3f,g).^[^ ^125^ ^]^ These reactive radiolysis products are chemically neutralized by graphene, mitigating electron‐beam‐induced damage. Therefore, biomolecules and other electron‐sensitive materials within GLCs exhibit improved stability compared to those in microfabricated SiN_*x*_ liquid cells. Additionally, graphene membranes exhibit high in‐plane thermal^[^ ^126^, ^127^ ^]^ and electrical conductivity,^[^ ^128^, ^129^ ^]^ facilitating efficient dissipation of accumulated heat and electrical charges caused by electron beam irradiation. This capability significantly reduces radiolysis‐induced artifacts, enabling more precise analysis of intrinsic nanoscale processes. Leveraging the advantages of graphene windows, GLCs enable structural characterization of electron‐beam‐sensitive materials, such as biological specimens^[^ ^130^, ^131^, ^132^, ^133^, ^134^, ^135^ ^]^ and polymers.^[^ ^136^, ^137^ ^]^ Furthermore, the inherent mechanical flexibility of graphene enables the formation of numerous isolated liquid pockets,^[^ ^138^ ^]^ providing multiple independent regions for observation. This design addresses the limitation of microfabricated SiN_*x*_ liquid cells, where the restricted number of liquid chambers limits the number of observations.

**Figure 4:**
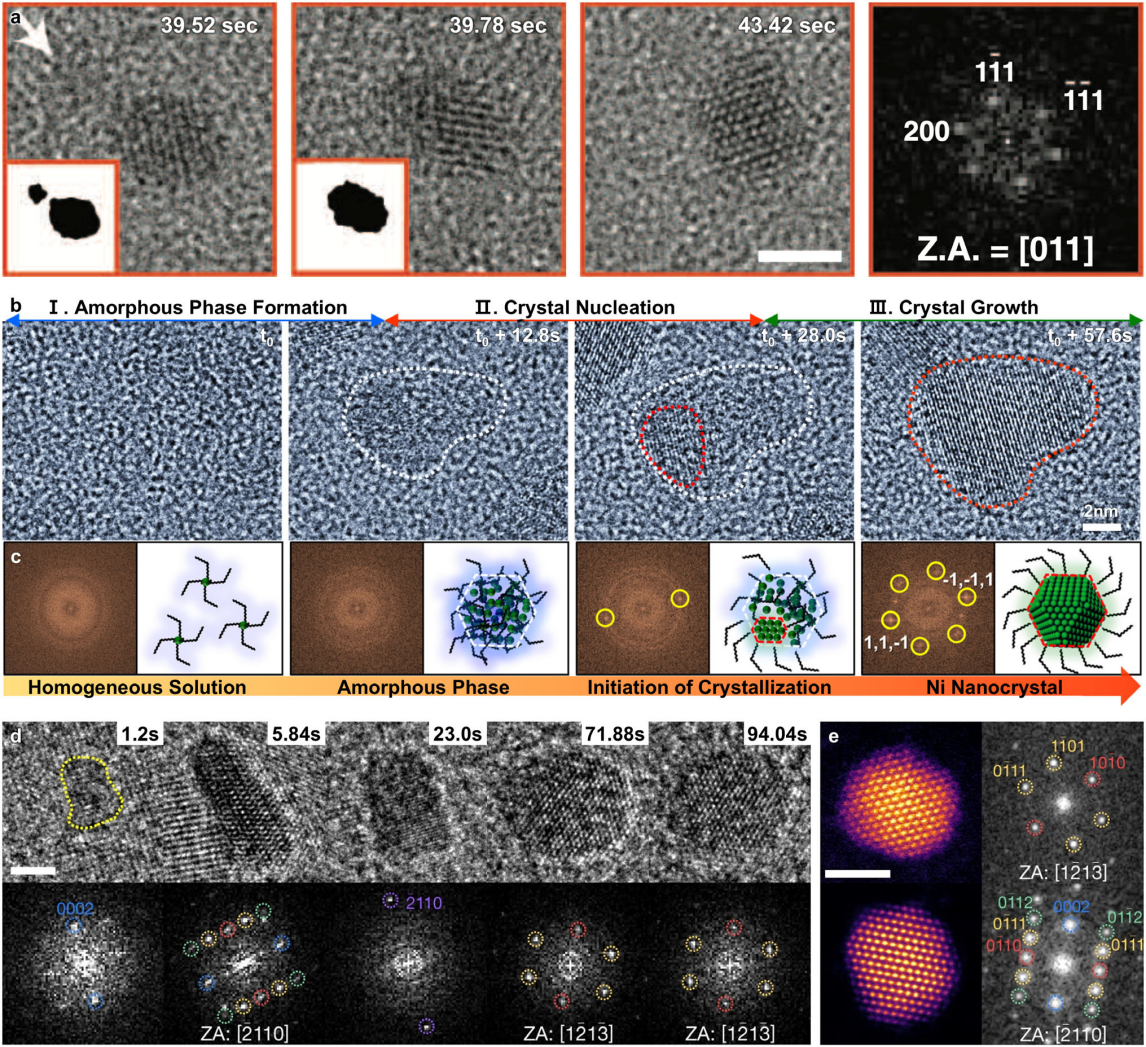
Liquid‐phase TEM studies of nanocrystal formation using veil‐type GLCs. a) TEM images showing oriented coalescence of two nanocrystals along the <111> direction, merging into a single face‐centered cubic nanocrystal. Scale bar: 2 nm. Reproduced with permission.^[^ ^91^ ^]^ Copyright 2012, AAAS. b) Time‐series TEM images revealing amorphous‐phase‐mediated crystallization. c) FFT images and schematic illustrations corresponding to each stage. Reproduced with permission.^[^ ^92^ ^]^ Copyright 2019, American Chemical Society. d) Time‐series TEM images of Pd nanoparticle growth at the high electron‐dose rate (≈1.0 × 10^4^ e^−^ Å^−2^ s^−1^), with corresponding FFT images. Scale bar: 2 nm. e) Ex situ high‐resolution STEM and FFT images of four particles generated at the electron‐dose rate of 675 e^−^ Å^−2^ s^−1^. Scale bar: 2 nm. Reproduced with permission.^[^ ^93^ ^]^ Copyright 2022, Springer Nature.

Based on the aforementioned advantages, nucleation and growth pathways of nanocrystals—widely used as catalysts^[^ ^139^, ^140^, ^141^, ^142^, ^143^, ^144^, ^145^, ^146^, ^147^, ^148^ ^]^ and in energy storage materials^[^ ^149^, ^150^, ^151^, ^152^ ^]^—have been extensively studied using veil‐type GLCs, revealing pathways beyond those predicted by classical nucleation theory. Typically, the nucleation process is triggered by electron beam‐induced reduction of metal precursors. Due to the high Z‐contrast of metal atoms, GLCs enable effective tracking of metal‐containing species during their nucleation and subsequent growth. One of the earlier studies by Yuk et al. revealed that Pt nanocrystal growth proceeded via both classical monomer‐by‐monomer attachment^[^ ^153^, ^154^ ^]^ and nanoparticle coalescence—a nonclassical growth pathway (**Figure** **4**a).^[^ ^91^ ^]^ This coalescence occurred through two mechanisms: 1) {111} mirror‐plane fusion and 2) twin boundary formation.

Recent studies using GLCs have further revealed intricate nonclassical growth pathways,^[^ ^155^, ^156^, ^157^, ^158^ ^]^ challenging classical crystallization theories and offering novel insights into nanoparticle growth mechanisms in liquids, enabled by the high spatial and temporal resolution of in situ GLC TEM. Yang et al. observed Ni nanocrystal crystallization pathways mediated by amorphous intermediates (Figure 4b,c).^[^ ^92^ ^]^ Instead of nucleating directly from the precursors, Ni precursors initially aggregated into amorphous intermediates, within which crystalline domains subsequently formed and grew. The transformation of the amorphous intermediate into crystalline nanocrystals sometimes involved the formation of multiple crystalline domains within a single amorphous intermediate, which can further transform into a single‐crystalline nanocrystal through dislocation relaxation.

Similarly, Dachraoui et al. identified less crystalline intermediate phases, referred to as “cluster‐clouds,” during the crystallization process.^[^ ^95^ ^]^ These cluster‐clouds formed within supersaturated precursor solutions and exhibited less crystalline features. Importantly, their growth mechanism differed from classical monomer‐attachment models; multiple cluster‐clouds coalesced and subsequently attached to growing crystal surfaces, while intrinsic structural relaxation involved reversible monomer attachment and detachment processes. Eventually, the initially polycrystalline nanocrystals transformed into single‐crystalline nanocrystals, which reflected complex mass transport dynamics between the solution, nuclei, and transient intermediates.

Moreover, metastable phases can be kinetically stabilized during the growth process by precisely controlling reaction conditions such as precursor concentrations. Hong et al. showed that sufficiently high hydrogen concentrations and limited availability of Pd precursors kinetically stabilized the metastable hexagonal close‐packed (HCP) PdH_*x*_ phase by suppressing its further growth and transformation into the thermodynamically stable face‐centered cubic (FCC) phase (Figure 4d,e).^[^ ^93^ ^]^ At lower hydrogen concentration and with abundant Pd precursors, the formation of the FCC phase was preferred.

**Figure 5:**
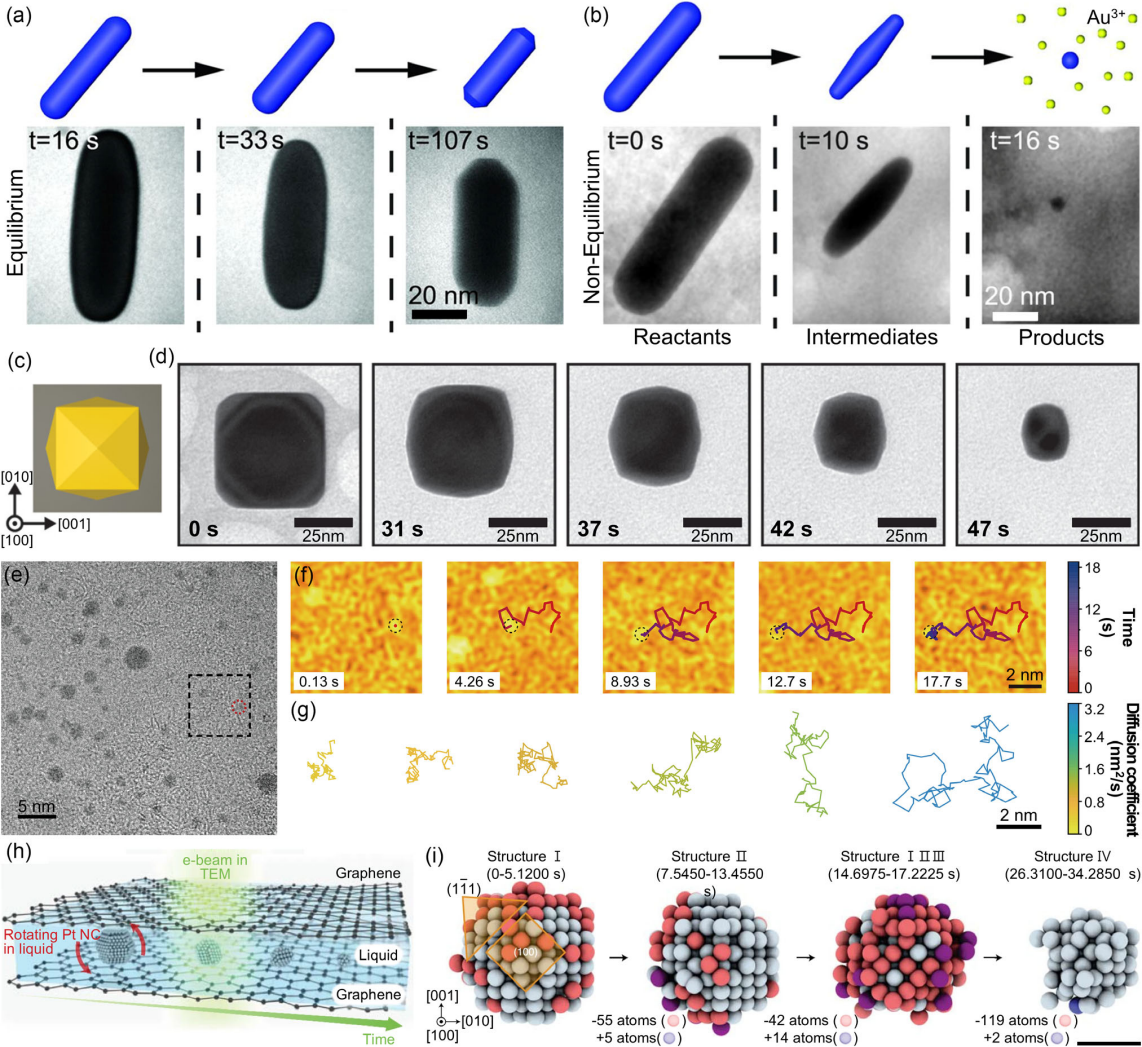
Liquid‐phase TEM studies of nanocrystal transformation and motion using veil‐type GLCs. a,b) Schematic illustration of the reaction pathway and selected TEM images showing a) the formation of near‐equilibrium nanorods that terminate with flat {100} tips and b) the transformation from rods to nonequilibrium ellipsoidal intermediates that terminate with sharp tips. Reproduced with permission.^[^ ^96^ ^]^ Copyright 2016, AAAS. c) Schematic illustration of a tetrahexahedron (THH, yellow) after anisotropic etching of a cube (gray). d) Time‐series TEM images showing etching from a cube to a THH. Reproduced with permission.^[^ ^97^ ^]^ Copyright 2018, American Chemical Society. e) Snapshot captured from in situ TEM movie showing Au nanoparticles dispersed in GLCs. f) False‐color TEM images of the region in (e) indicated with dotted line, visualizing the random motion of nanoparticles. g) Trajectories of individual Au nanoparticles, color reflecting diffusion coefficient. Reproduced with permission.^[^ ^158^ ^]^ Copyright 2021, AAAS. h) Schematic of Pt nanocrystal etching in GLCs. i) Time‐resolved 3D atomic maps showing removed atoms (red), rearranged atoms (blue), and atoms that were rearranged in previous maps and removed in subsequent stages (purple). Scale bar: 1 nm. Reproduced with permission.^[^ ^152^ ^]^ Copyright 2025, Springer Nature.

In addition to nucleation and growth processes, various dynamic processes including etching^[^ ^96^, ^97^, ^98^, ^99^, ^100^, ^101^, ^102^ ^]^ and motions^[^ ^91^, ^159^, ^160^, ^161^, ^162^, ^163^ ^]^ of nanoparticles have been directly visualized using GLCs. Interestingly, shape transformations during etching generated intermediate states with diverse morphologies. Ye et al. investigated the oxidative dissolution of Au nanoparticles in an aqueous solution,^[^ ^96^ ^]^ introducing FeCl_3_ as the primary etchant. Reactive radiolysis products generated by electron beam irradiation triggered nanoparticle etching. Under equilibrium conditions at low FeCl_3_ concentrations, Au nanoparticles exhibited relatively slow etching rates, allowing structural relaxation and the formation of stable, low‐energy crystal facets (**Figure** **5**a). In contrast, under nonequilibrium conditions, 1D Au nanoparticles developed transient sharp‐tipped morphologies, indicating limited structure relaxation during rapid etching (Figure 5b). Moreover, Hauwiller et al. showed that Au nanocubes and rhombic dodecahedra transformed into kinetically stable tetrahexahedra with high‐index {hk0} facets under nonequilibrium conditions (Figure 5c,d), which are kinetically favored despite being thermodynamically less stable.^[^ ^96^, ^97^ ^]^

Veil‐type GLCs effectively enabled the observation of nanoparticle motions and interparticle interactions within liquids by minimizing charging effects from supporting substrates. Kang et al. investigated the thermal motions and coalescence behaviors of ligand‐passivated colloidal Au nanoparticles (Figure 5e–g).^[^ ^159^ ^]^ The measured diffusion coefficients were smaller than those predicted by the Stokes–Einstein relation, despite negligible electron beam influence. Instead, Au nanoparticles exhibited Fickian diffusion within nanoconfined liquid pockets of GLCs. Additionally, nanoparticle displacement distributions deviated from Gaussian distributions, reflecting fluctuations in diffusivity. Interestingly, nanoparticles formed transient complexes upon diffusive contact before coalescing. After complex formation, the diffusion coefficients decreased, indicating an increased hydrodynamic size. This two‐step coalescence mechanism involving intermediate complex formation provided a more accurate explanation of the experimental observations compared to a single‐step direct coalescence pathway.

Recent advancements in GLCs have leveraged the dynamic motions of colloidal nanoparticles in liquids to achieve atomic‐scale 3D reconstructions.^[^ ^160^, ^161^, ^162^, ^163^ ^]^ Conventional TEM‐based tomography techniques can reconstruct 3D nanocrystal structures; however, these approaches have inherent limitations. Specifically, conventional methods rely on image acquisition under vacuum conditions, directly exposing nanoparticles to high‐energy electron beams and necessitating extensive specimen tilting, which limits the structural analysis of nanoparticles immobilized on substrates. In contrast, high‐resolution GLC‐TEM enables the direct imaging of nanoparticle structures from multiple angles by tracking their Brownian motion in liquid. This approach allows the acquisition of multiple 2D TEM images from various nanoparticle orientations, facilitating accurate 3D reconstruction.

Recent methodological developments have further advanced this capability, enabling detailed analysis of atomic arrangements in nanocrystals^[^ ^162^, ^163^ ^]^ and real‐time tracking of atomic‐scale structural evolution during oxidative etching processes (Figure 5h,i).^[^ ^163^ ^]^ A self‐supervised denoising framework combined with advanced reconstruction algorithms, applied to millisecond‐resolution imaging of Pt nanoparticle etching trajectories, enabled accurate tracking of the structural evolution of individual nanocrystals at 3D atomic resolution. Notably, a complete dissolution trajectory was observed, revealing a transition from an FCC crystalline structure to a disordered state as particle size decreased, indicating stabilization of the disordered state at the nanoscale.^[^ ^164^ ^]^ Atomic‐scale reconstruction further demonstrated preferential etching at surface defect sites such as kinks and steps, accompanied by structure rearrangements that decreased FCC‐like symmetry while simultaneously increasing HCP‐like characteristics. These findings underscore the capability of in situ liquid‐phase TEM using GLCs to perform real‐time atomic‐scale structure reconstructions, providing insights into nanoscale metastable intermediates distinct from those observed in bulk structural transformations.

Additionally, GLCs have enabled the direct visualization of dynamic behaviors of surface ligands on colloidal nanocrystals.^[^ ^165^ ^]^ Pedrazo‐Tardajos et al. visualized hexadecyltrimethylammonium bromide (CTAB) ligands adsorbed on gold nanorods with high imaging contrast. The CTAB ligand shell exhibited heterogeneous thickness distributions depending on liquid pocket volumes, with notably thicker layers along lateral facets compared to nanorod tips. Notably, the dynamic attachment of CTAB micelles from the surrounding solution onto the crystal surfaces highlighted the mobile and dynamic characteristics of surface ligands, offering insights into ligand‐crystal surface interactions.

Despite these significant achievements, veil‐type GLCs exhibit several limitations. The liquid pockets spontaneously form with random size and thickness,^[^ ^91^ ^]^ requiring considerable effort to identify and select suitable regions for studying reaction pathways. Additionally, inconsistencies in liquid confinement geometry across individual pockets,^[^ ^91^, ^159^ ^]^ as well as significant variation in the local liquid thickness within a single liquid pocket,^[^ ^166^ ^]^ complicate quantitative analysis and reduce the reproducibility of observations. Furthermore, sealing induced by evaporation during liquid cell fabrication can significantly alter the concentration of encapsulated solutions from the initially intended values. These challenges have driven recent efforts toward developing advanced GLC configurations to enhance control over liquid encapsulation and achieve higher experimental reproducibility.

## 3 Well‐Type GLCs

Although veil‐type GLCs offer high imaging resolution and relatively straightforward fabrication by simply sandwiching liquid samples between two atomically thin graphene sheets, they exhibit limitations in experimental reproducibility. Specifically, liquid pockets form at random locations, exhibiting considerable variability in their geometrical configurations, including thickness.^[^ ^91^ ^]^ Such inherent variability complicates systematic and reproducible observations of liquid‐phase reaction pathways, as local reaction conditions—such as solution volume and nanoparticle concentration—may significantly differ between individual observations, potentially influencing reaction kinetics. Therefore, advanced GLC designs that enable precise geometric control over encapsulated liquids—while preserving the high spatial resolution and minimal background scattering afforded by atomically thin graphene windows—are required.

Well‐type GLCs have thus emerged as an effective alternative to veil‐type designs, providing chambers with well‐defined geometries with controlled volumes, which significantly reduce variations in local liquid thickness and confinement, thereby ensuring highly reproducible experimental conditions for in situ liquid‐phase TEM studies. In this configuration, substrates featuring regularly spaced arrays of holes, referred to as “wells,” are prepared through microfabrication techniques. Spacer membranes for these well arrays include SiN_*x*_,^[^ ^167^, ^168^, ^169^ ^]^ hexagonal boron nitride (hBN),^[^ ^170^ ^]^ or anodic aluminum oxide (AAO).^[^ ^171^, ^172^ ^]^ Following microfabrication of the hole arrays, graphene serves as the atomically thin sealing membrane on both sides of the spacer, forming electron‐transparent and liquid impermeable windows.^[^ ^90^, ^173^ ^]^ Because graphene adheres tightly to the micropatterned spacer membrane through van der Waals interactions, the enclosed liquid is effectively confined in each individual well, and its thickness is governed primarily by the spacer membrane thickness. Arrays of uniformly dimensioned wells enable multiple observations of nanoscale processes under nearly identical experimental conditions. This design contrasts with veil‐type GLCs, where liquid pockets form irregularly. The precisely controlled geometry—including defined volume, lateral dimensions, and thickness—of liquid pockets in well‐type GLCs facilitates reproducible experimental observations and enables quantitative analysis of reaction kinetics within nanoconfined liquid environments. Additionally, physical isolation of each well ensures that electron‐beam‐induced events or local environmental changes occurring within one well do not influence the reaction conditions within neighboring wells.

**Figure 6:**
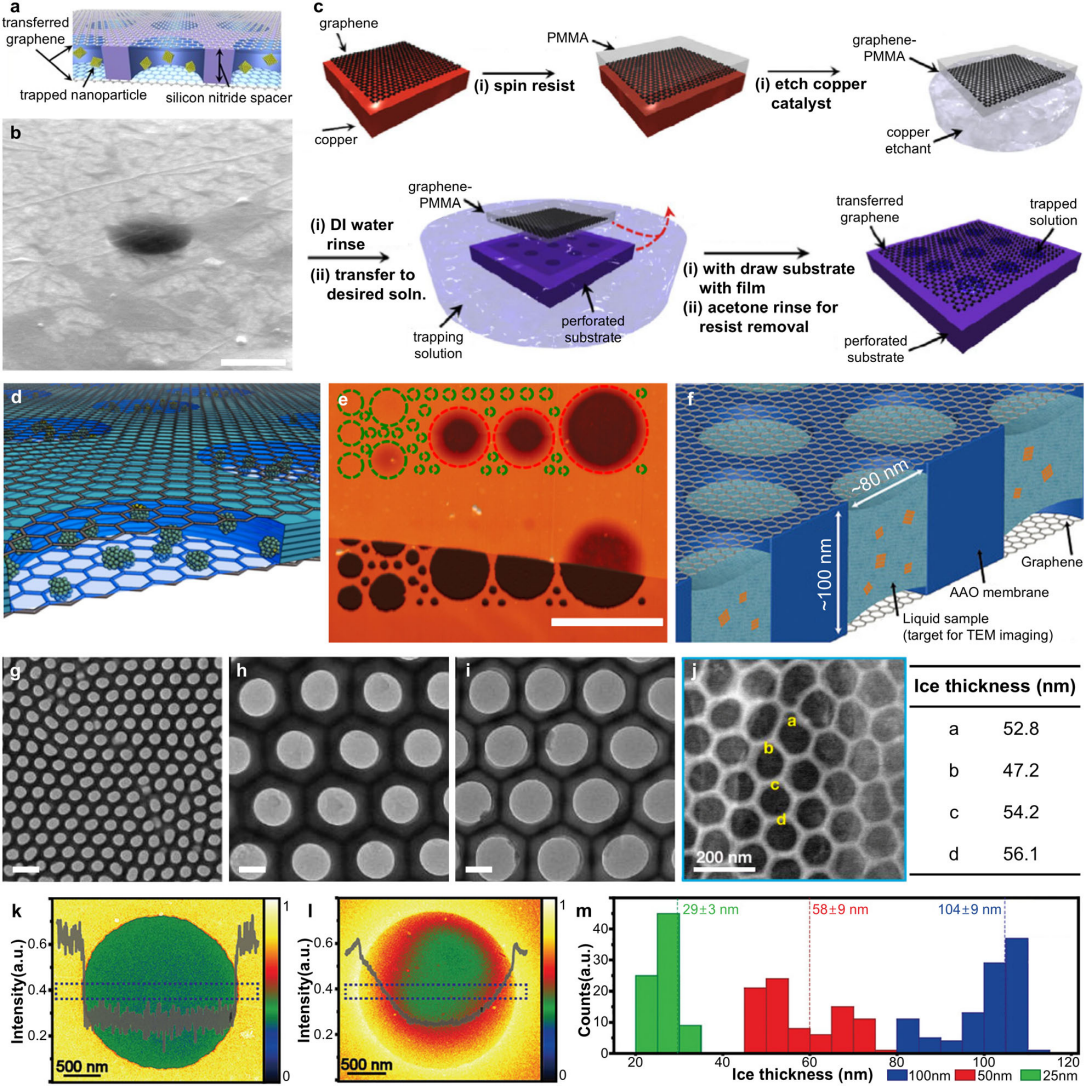
Structure and fabrication of well‐type GLCs. a) Schematic of well‐type GLC. b) SEM image of well‐type GLC. Scale bar: 500 nm. c) Schematic illustration of CVD‐graphene transfer onto a substrate with patterned cavities. Reproduced with permission.^[^ ^167^ ^]^ Copyright 2016, Wiley‐VCH. d) Illustration of an engineered GLC loaded with nanocrystals. e) AFM map on a Si wafer, indicating wells with (green) and without liquid (red). The hBN spacer is visible below the graphene edge. Scale bar: 2 μm. Reproduced with permission.^[^ ^170^ ^]^ Copyright 2018, American Chemical Society. f) Schematic of nanochamber arrays with an AAO spacer. g–i) TEM images of AAO membranes with nanopore diameters of g) 80 nm, h) 260 nm, and i) 340 nm and corresponding interpore spacings of g) 125 nm, h) 450 nm, and i) 450 nm, respectively. Scale bars: 200 nm. Reproduced with permission.^[^ ^171^ ^]^ Copyright 2020, Wiley‐VCH. j) Cryogenic STEM images of multichamber GLCs with a 60‐nm‐thick AAO membrane (left) and the corresponding ice‐thickness map (right). Reproduced with permission.^[^ ^172^ ^]^ Copyright 2020, American Chemical Society. k–m) Vitreous‐ice thickness analysis on micropatterned supports with different depths. Energy‐filtered TEM (EFTEM) images and line scan for k) a 50 nm device, l) a conventional holey carbon grid, and m) thickness histograms for 25,50, and 100 nm devices. Reproduced with permission.^[^ ^168^ ^]^ Copyright 2021, Wiley‐VCH.

Rasool et al. fabricated well‐type GLCs with holes ≈400 nm in diameter and 200 nm in depth (**Figure** **6**a,b), encapsulating liquid volumes in the tens of attoliters range.^[^ ^167^ ^]^ Hole arrays were micropatterned using focused ion beam (FIB). Subsequently, CVD‐grown graphene was transferred from a Cu substrate onto a micropatterned SiN_*x*_ membrane using polymethyl methacrylate (PMMA) as a support layer to seal the liquid wells. After etching the Cu substrate with sodium persulfate, the graphene‐PMMA films were transferred onto the patterned SiN_*x*_ membrane, and the PMMA was subsequently removed with acetone to improve graphene‐SiN_*x*_ adhesion. By sandwiching liquid droplets between graphene sheets positioned on opposite sides of the SiN_*x*_ membrane, enclosed liquid wells were successfully formed (Figure 6c). Although this method provided well‐defined geometries of liquid wells and leveraged the inherent advantages of graphene windows, encapsulated liquids evaporated or leaked after 10 min of electron beam irradiation.^[^ ^167^ ^]^ These limitations underscored the necessity for improved sealing techniques to prevent leakage due to evaporation.

Kelly et al. introduced an hBN membrane as a spacer, with micropatterned wells ranging from 100 nm to 1.5 μm in diameter (Figure 6d,e).^[^ ^170^ ^]^ The thickness of hBN can be precisely controlled from a few atomic layers up to 2 μm, thereby enabling accurate modulation of the encapsulated liquid thickness. Utilizing a thin and flat hBN membrane as a spacer—exhibiting strong van der Waals interactions with graphene—enabled robust sealing and effective isolation of individual wells, even after the rupture of other liquid wells. The absence of liquid leakage was confirmed even after 26 h of vacuum exposure during repeated atomic force microscopy (AFM) measurements. Furthermore, the use of bi‐ or trilayer graphene as sealing windows significantly increased the fabrication success rate, while the inherently high basal‐plane thermal conductivity of hBN^[^ ^174^ ^]^ facilitated efficient heat dissipation. Consequently, no leakage of liquids was observed even during heating at 120 °C in liquid cells encapsulating a water/isopropanol mixture.

Lim et al. introduced an AAO membrane with a thickness of ≈100 nm as a spacer, featuring well‐ordered nanopores (Figure 6f).^[^ ^171^ ^]^ The nanopore diameter can be precisely tuned from 80 to 340 nm (Figure 6g–i), providing control over the encapsulated liquid volume within each individual well. During the anodization process, the AAO membrane developed a uniform and highly ordered nanoporous structure, with nanopores forming simultaneously throughout the AAO membrane. Approximately 4.5 × 10^7^ nanopores were fabricated within a 3 mm‐diameter AAO membrane using this method, with each nanopore functioning as an individual liquid well. This nanopore density surpasses that previously achieved with other well‐type GLC configurations,^[^ ^167^, ^171^ ^]^ significantly increasing the opportunities for quantitative and consistent analyses of liquid‐phase reaction mechanisms. Furthermore, the pore size can be regulated by adjusting the anodization parameters. Subsequently, Bae et al. further advanced the AAO‐integrated GLC design by reducing the chamber thickness to ≈50–60 nm (Figure 6j), with an average liquid thickness of 39.8 ± 11.5 nm.^[^ ^172^ ^]^

The well‐type GLC design—consisting of a micropatterned spacer membrane enclosed by graphene sheets—has also been applied to cryogenic electron microscopy (cryo‐EM) for more reliable data acquisition and reproducible sample preparation. Although cryo‐EM is highly effective for resolving structures of electron‐beam‐sensitive materials,^[^ ^175^, ^176^, ^177^, ^178^, ^179^ ^]^ conventional sample preparation methods often lack precise control over ice thickness, an important factor affecting both imaging resolution and the signal‐to‐noise ratio. Excessively thick ice layers cause significant electron scattering, necessitating finely controlled and uniform ice layers. Kan et al. utilized the well‐type GLC design in cryo‐EM using a micropatterned SiN_*x*_ membrane sealed with 4 nm‐thick graphene oxide (GO) windows, which were fabricated through drop‐casting GO solution onto the patterned substrate.^[^ ^168^ ^]^ The ice layer encapsulated with GO exhibited significantly enhanced thickness uniformity (Figure 6k) compared to samples prepared on conventional holey carbon film grids, which showed inhomogeneous thickness distributions (Figure 6l). Ice layer thickness within each micropatterned hole was controlled to ≈25, 58, and 104 nm (Figure 6m), accurately aligning with target thicknesses of 25, 50, and 100 nm, respectively. This enabled reproducible and high‐resolution imaging of biomaterials.

**Figure 7:**
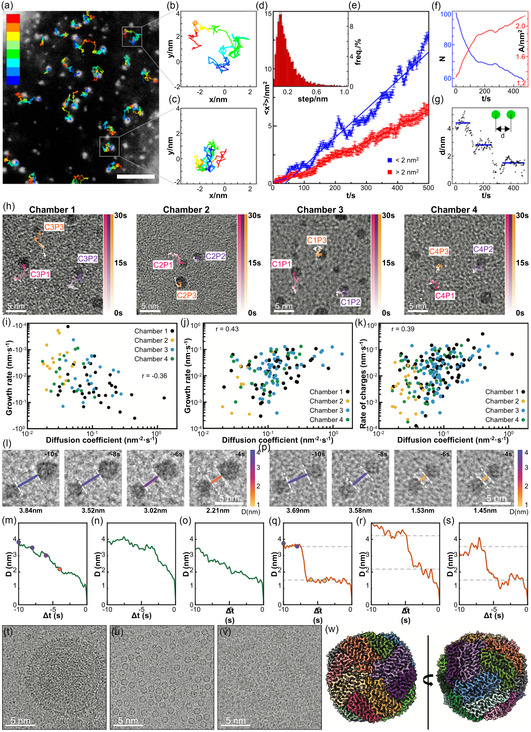
Quantitative analysis of nanoscale processes in well‐type GLCs. a) Snapshot from a real‐time movie with individual trajectories superimposed. Scale bar: 10 nm. b,c) The movement trajectories of single nanocrystals with mean projected areas of b) 1.3 nm^2^ and c) 1.7 nm^2^. d) The relative frequency of frame‐to‐frame step displacements for all tracked particles. e) Time‐dependent mean square displacements for nanocrystals with different sizes. f) The number of particles (blue) and average projected area of nanocrystals (red) captured per unit time. g) Time‐dependent interparticle distance between two nanocrystals before coalescence. Reproduced with permission.^[^ ^170^ ^]^ Copyright 2018, American Chemical Society. h) TEM images showing the diffusion trajectories of three selected nanoparticles from four separate nanochambers. i–k) Diffusion‐coefficient‐dependent (i) dissolution rate, (j) growth rate, and (k) absolute rate of change. Reproduced with permission.^[^ ^171^ ^]^ Copyright 2020, Wiley‐VCH. l–s) Time‐series TEM images (upper rows) and corresponding gap‐distance trajectories (lower rows) for AuNPs in l–o) CTAB and p–s) mixed‐surfactant systems before nanoparticle contact. Reproduced with permission.^[^ ^172^ ^]^ Copyright 2020, American Chemical Society. t–v) Cryo‐EM images of biomolecules captured in micropatterned wells of different depths: t) HIV‐1 (≈100 nm) in a 100 nm‐thick hole, u) apoferritin (470 kDa) in a 50 nm‐thick hole, and v) aldolase (157 kDa) in a 25 nm‐thick hole. w) Cryo‐EM map showing 3D structure of horse apoferritin with 3.06 Å resolution. Reproduced with permission.^[^ ^168^ ^]^ Copyright 2021, Wiley‐VCH.

By encapsulating discrete liquid volumes within geometrically uniform wells, well‐type GLCs facilitate consistent quantitative analyses of nanoscale dynamic processes. Rasool et al. revealed the motions of nanoparticles within confined liquid wells.^[^ ^167^ ^]^ Initially, tungsten nanoparticles were formed by electron‐beam‐induced reduction of tungsten hexachloride in solution.^[^ ^170^, ^180^ ^]^ After nucleation, nanoparticles exhibited diffusive motion, which was systematically tracked using time‐series high‐angle annular dark‐field scanning transmission electron microscopy (HAADF‐STEM) imaging at a temporal resolution of 2.5 s (**Figure** **7**a–c). Quantitative analysis involved ≈5000 measurements of the mean square displacement ⟨x^2^⟩ as a function of time, confirming that tungsten nanoparticles followed 2D Brownian motion described by ⟨x^2^⟩ = 4*Dt*, where *D* represents the diffusion coefficient. Larger nanoparticles exhibited smaller *D* values compared to their smaller counterparts, suggesting size‐dependent mobility (Figure 7d,e). Notably, *D* measured within these well‐type GLCs was significantly lower than those values previously reported for microfabricated SiN_*x*_ liquid cells and bulk liquid environments.^[^ ^181^, ^182^, ^183^ ^]^ This reduced mobility was primarily attributed to nanoparticle–graphene interfaces, interactions with neighboring nanoparticles, and residual hydrocarbon contamination. Further analyses revealed that nanoparticle growth was accompanied by a progressive decrease in nanoparticle number, indicating that Ostwald ripening and particle coalescence occurred (Figure 7f). Particle coalescence is typically initiated with the formation of nanoparticle pairs, characterized by correlated movements prior to direct contact. These nanoparticle pairs maintained consistent center‐to‐center distances for hundreds of seconds before merging (Figure 7g).

Furthermore, Lim et al. statistically investigated the growth and motions of gold nanoparticles in liquid through repeated observations under uniform electron beam conditions across multiple liquid wells.^[^ ^171^ ^]^ Multiple nanoparticles observed within separate wells were independently tracked (Figure 7h), confirming Brownian motion characterized by diffusion coefficients ranging from 1.9 × 10^−^^2^ to 9.6 × 10^−^^2^ nm^2^ s^−^^1^.^[^ ^184^ ^]^ During the early growth stage, the average particle size initially decreased slightly due to the continuous nucleation of small nanoclusters. This stage was subsequently followed by a decrease in particle numbers driven by coalescence. These observations indicated that both monomer attachment and particle coalescence mechanisms contributed significantly to the growth of colloidal nanoparticles. Notably, repeated multichamber observations revealed a positive correlation between nanoparticle diffusion coefficients and the rates of particle dynamics, including growth and dissolution (Figure 7i–k). This correlation highlights the necessity of large‐scale data collection for accurately elucidating nanoscale processes.

Quantitative in situ TEM analyses further provided valuable insights into nanoparticle coalescence processes. Careful consideration of surface ligands is crucial, as they significantly modulate interparticle interactions and consequently influence nanoparticle growth pathways.^[^ ^185^ ^]^ Specifically, interparticle forces—particularly electrostatic interactions—are primarily governed by surface ligands,^[^ ^186^ ^]^ emphasizing the importance of understanding ligand effects during nanoparticle coalescence‐mediated growth. Bae et al. investigated ligand effects on nanoparticle coalescence by examining two distinct ligand systems: 1) gold nanoparticles capped only with CTAB, forming interdigitated bilayer structures,^[^ ^187^ ^]^ and 2) gold nanoparticles capped with mixed ligands comprising CTAB and octylamine in a 3:1 molar ratio.^[^ ^172^ ^]^ More than 40 nanoparticle pairs were analyzed to derive conclusions. Nanoparticles capped exclusively with CTAB exhibited a gradual and continuous reduction in interparticle distances, indicating a progressive nanoparticle approach toward coalescence (Figure 7l–o). Upon reaching an interparticle gap of ≈1 nm, nanoparticle pairs showed abrupt “jump‐to‐contact” behavior. In contrast, nanoparticles capped with mixed ligands showed an abrupt decrease in interparticle distance from ≈3.5 nm to ≈1.5 nm (Figure 7p–s). Molecular dynamics (MD) simulations corroborated these experimental findings, revealing that the introduction of octylamine disrupted the formation of interdigitated bilayer structures of CTAB, thereby significantly enhancing ligand mobility. This increased mobility effectively reduced enthalpic barriers, facilitating rapid nanoparticle approach and subsequent coalescence.

Moreover, well‐type GLCs have been further applied to the 3D structural reconstruction of biomolecules, advancing cryo‐EM techniques.^[^ ^168^ ^]^ Excessive ice thickness in cryo‐EM increases background electron scattering, thereby reducing imaging contrast.^[^ ^188^ ^]^ By precisely controlling ice thickness in well‐type GLCs, ice‐layer thickness was optimized to the dimensions of the samples, ensuring improved imaging contrast without affecting the intrinsic structures of embedded biomolecules. Consequently, structures of biomolecules—such as HIV‐1 particles (≈100 nm), apoferritin (470 kDa), and aldolase (157 kDa)—were successfully identified using micropatterned chips with controlled depths of 100 nm, 50 nm, and 25 nm, respectively (Figure 7t–w). Specifically, apoferritin was reconstructed with near‐atomic resolution (3.06 Å), validating the effectiveness of well‐type GLC configurations for high‐resolution cryo‐EM imaging.

Despite significant advances in quantitative characterization enabled by geometrically well‐defined liquid chambers, well‐type GLCs present inherent structural limitations. First, strong van der Waals forces between graphene windows, combined with hydrophilic interactions between liquid and spacer membranes (e.g., SiN_*x*_, hBN, and AAO membranes), induce liquids being preferentially confined primarily near spacer edges. This confinement forms “ring‐type” liquid cells rather than uniformly distributed liquid layers across the entire well region.^[^ ^189^ ^]^ To prevent the formation of ring‐type liquid distributions, precise dimensional control of both the diameter (d) and height (h) of the liquid chambers is required, where the transition point is observed at *d*/*h* ratio of 4. Furthermore, well‐type GLCs typically encapsulate only a single liquid solution at a time, complicating the manipulation of reaction conditions and the introduction of reactants. These constraints pose significant challenges for in situ TEM studies of complex, multistep chemical reaction pathways that require manipulation of reaction conditions.

Additionally, the sophisticated microfabrication processes involved in the fabrication of well‐type GLCs—such as lithography, wet etching, and FIB milling—lead to an increase in manufacturing costs. These intricate fabrication steps potentially limit large‐scale production and broader accessibility. Consequently, further technological advancements are required to develop facile fabrication methods and to extend the applicability of GLC technology.

## 4 Liquid‐Flowing‐Type GLCs

Liquid‐flowing‐type GLCs constitute a significant methodological advancement for in situ liquid‐phase TEM, enabling precise manipulation of reaction conditions through the controlled introduction of reactants into the liquid chamber. Conventional static GLC designs, including veil‐type and well‐type GLCs, confine a preloaded liquid sample between two graphene sheets, inherently restricting the on‐demand addition of external chemicals. Consequently, these static designs limit observations primarily to reactions triggered by electron beam irradiation. Additionally, static GLC configurations require preloading and premixing of all reactants during sample preparation, thus preventing real‐time observations of initial reaction stages.

Microfabricated SiN_*x*_ liquid cells incorporate fluidic channels designed for modulation of chemical environments through chemical addition,^[^ ^63^, ^71^, ^76^, ^77^ ^]^ substantially broadening the scope of observable nanoscale processes. The integration of fluidic channels into the liquid chambers facilitates real‐time modification of solution composition and extends observation times by continuously circulating the solution through connections between the chambers and external inlets, thereby reducing the accumulation of radiolysis‐induced byproducts. Furthermore, this continuous liquid flow effectively mitigates gas bubble accumulation, maintaining stable imaging conditions.^[^ ^190^ ^]^ However, despite these advantages, SiN_*x*_ liquid‐flowing‐type cells exhibit limitations due to membranes that are typically tens of nanometers thick. These relatively thick imaging windows inevitably increase electron scattering and induce significant charging artifacts, presenting considerable challenges for high‐resolution imaging. Liquid‐flowing‐type GLCs directly address these issues by utilizing atomically thin graphene membranes as imaging windows, offering superior electron transparency, minimal charging effects under electron irradiation, and scavenging effect of reactive radiolysis products. Consequently, graphene membranes enhance the spatial resolution achievable in liquid‐phase TEM compared to SiN_*x*_‐based liquid‐flowing‐type cells.

**Figure 8:**
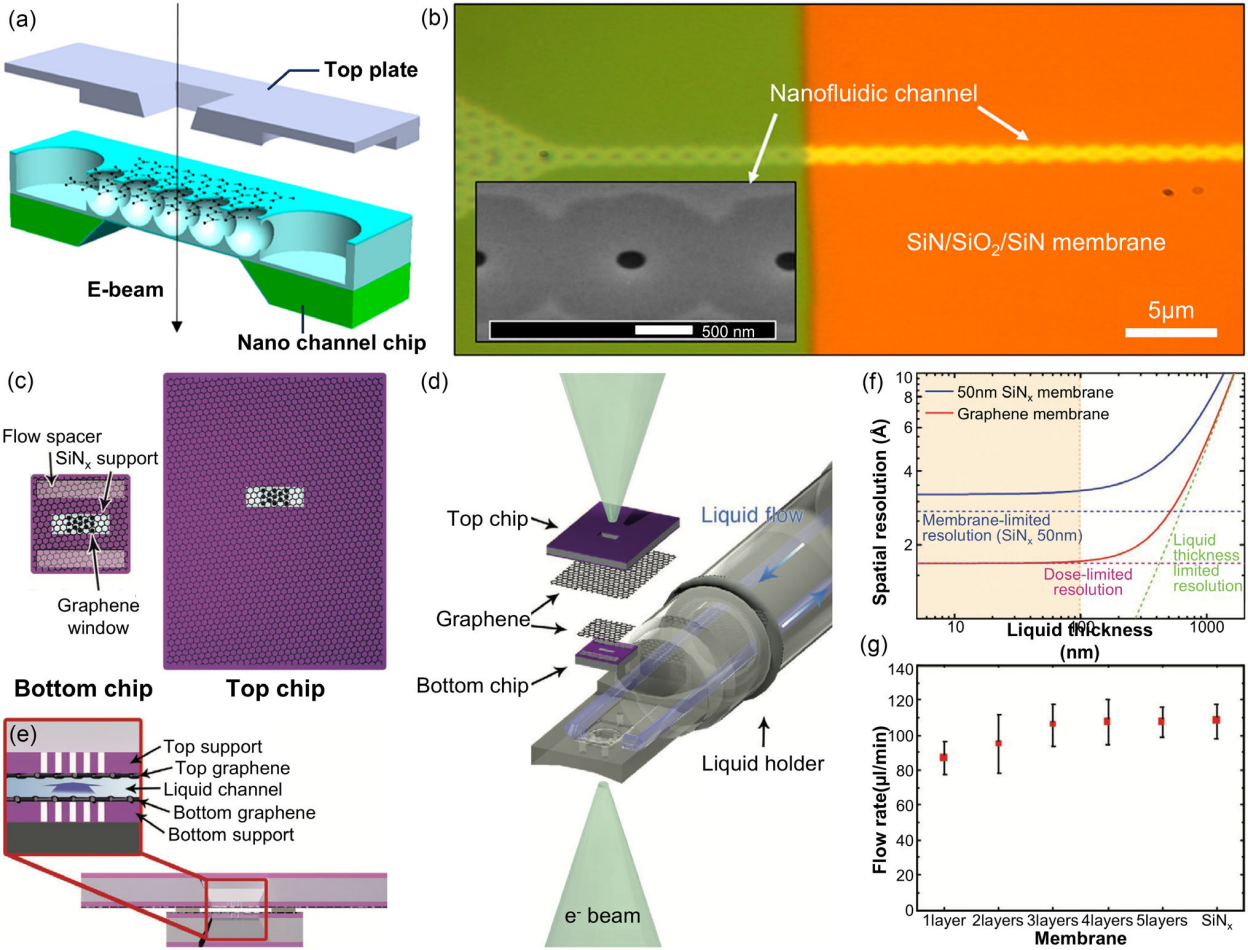
Structure and fabrication of liquid‐flowing‐type GLCs. a) Cross‐sectional schematic of the graphene flow cell showing the unassembled nanochannel chip and top plate. b) Optical image showing a nanofluidic channel fabricated by the selective removal of SiO_2_ from a Si_3_N_4_/SiO_2_/Si_3_N_4_ stack. Reproduced with permission.^[^ ^191^ ^]^ Copyright 2020, American Chemical Society. c) Top‐view image of the separated liquid‐flowing graphene chip showing the top chip and bottom chip with spacers. A graphene window covers the perforated SiN_*x*_ membranes. d) Schematic of liquid‐flow graphene chips in a liquid‐phase TEM holder, showing flow inlet and outlet. e) Cross‐section of the assembled chip, where the flow channel is formed between the stacked chips. f) Simulated spatial resolution with graphene membrane and a 50 nm‐thick SiN_*x*_ membrane at an electron dose of 1 × 10^5^ e^−^ Å^−2^. g) Maximum sustainable flow rate for liquid‐flow graphene chips and standard SiN_x_ chips. Reproduced with permission.^[^ ^194^ ^]^ Copyright 2021, Wiley‐VCH.

Dunn et al. developed advanced liquid‐flowing‐type GLCs that overcome the inherent limitations of static GLCs by enabling the on‐demand introduction of target solutions.^[^ ^191^ ^]^ Their liquid cell design featured a nanochannel‐integrated bottom chip consisting of a monolithically fabricated stack of Si_3_N_4_/SiO_2_/Si_3_N_4_ layers, with precisely controlled thicknesses of 14 nm, 75 nm, and 7 nm, respectively. Internal nanochannels with a width of ≈2 μm were fabricated via lithographic patterning and subsequent acid‐etching of the SiO_2_ layer. These nanochannels formed interconnected cavities sealed by graphene windows, providing multiple independent imaging regions and reducing interruption of experiments caused by potential channel blockages (**Figure** **8**a,b). The bottom part of the cell could be sealed using either graphene or an ultrathin Si_3_N_4_ membrane.

Moreover, membrane bulging is often observed in well‐type GLCs and SiN_*x*_‐based liquid cells due to pressure differentials across the imaging windows. Excessive deformation of membranes alters the liquid thickness distribution, thereby degrading both imaging contrast and achievable resolution.^[^ ^192^, ^193^ ^]^ Notably, in liquid‐flowing‐type GLCs, such bulging is effectively suppressed by designing the exposed aperture size (≈200 nm) to be substantially smaller than the internal cavity dimensions (≈2 μm), underscoring the critical role of liquid cell design for reducing bulging.

Koo et al. further advanced liquid‐flowing‐type GLC technology by integrating graphene membranes for both top and bottom imaging windows, significantly improving the achievable imaging resolution.^[^ ^194^ ^]^ Their liquid‐flowing‐type GLC structure comprises a pair of MEMS‐fabricated SiN_*x*_ chips, each incorporating graphene imaging windows and hole‐patterned apertures, designed to fit standard liquid‐flow TEM holders (Figure 8c–e). This design maintains mechanical rigidity through robust SiN_*x*_ supports, while graphene windows provide superior electron transparency, thereby improving imaging contrast for samples within liquid media. The improved spatial resolution achievable with liquid‐flowing‐type GLCs was quantitatively analyzed using parameters including Scherzer resolution (*d*_s_), chromatic aberration‐dependent resolution (*d*_c_), dose‐limited resolution (*d*_SNR_), and beam blurring‐limited resolution (*d*_b_). Collectively, these parameters define the resolution limit of liquid‐phase TEM imaging (*d*_TEM_), as represented by the equation:^[^ ^194^, ^195^ ^]^

(1) $$d_{\text{TEM}}=\sqrt{d_{s}^{2}+d_{c}^{2}+d_{\text{SNR}}^{2}+d_{b}^{2}}$$

Notably, for liquid layers thinner than 100 nm, graphene membranes significantly enhance *d*_TEM_ relative to SiN_*x*_ membranes by reducing *d*_c_ and *d*_b_. Consequently, the resolution under these conditions becomes predominantly limited by *d*_SNR_ (Figure 8f). Additionally, careful control of membrane deformation and liquid thickness remains critical for achieving atomic‐scale imaging. Membrane bulging substantially increases electron scattering, thereby degrading attainable spatial resolution. Computational simulations showed that increasing SiN_*x*_ support thickness effectively mitigates membrane bulging. Specifically, a SiN_*x*_ membrane thickness of ≈300 nm was demonstrated to significantly reduce membrane deflection to ≈22 nm. This controlled deflection facilitated atomic‐resolution imaging by precisely maintaining the liquid layer below 100 nm,^[^ ^195^ ^]^ which is achievable with 30 nm‐thick spacers. Furthermore, graphene membranes exhibited comparable pressure tolerance to that of 50 nm‐thick SiN_*x*_ membranes, enduring pressures of ≈3–6 bar and 4–10 bar, respectively. Moreover, graphene membranes showed remarkable mechanical resilience under laminar flow‐induced stresses, enduring liquid flow rates up to 80 μL min^−1^ for single‐layer graphene and exceeding 120 μL min^−1^ for multilayer graphene, indicating performance comparable to that of SiN_*x*_ membranes (Figure 8g). These advantages derived from the design of liquid‐flowing‐type GLCs represent significant improvements over conventional liquid‐phase TEM methodologies, fundamentally expanding the capabilities and application scope of in situ liquid‐phase imaging and analysis.

**Figure 9:**
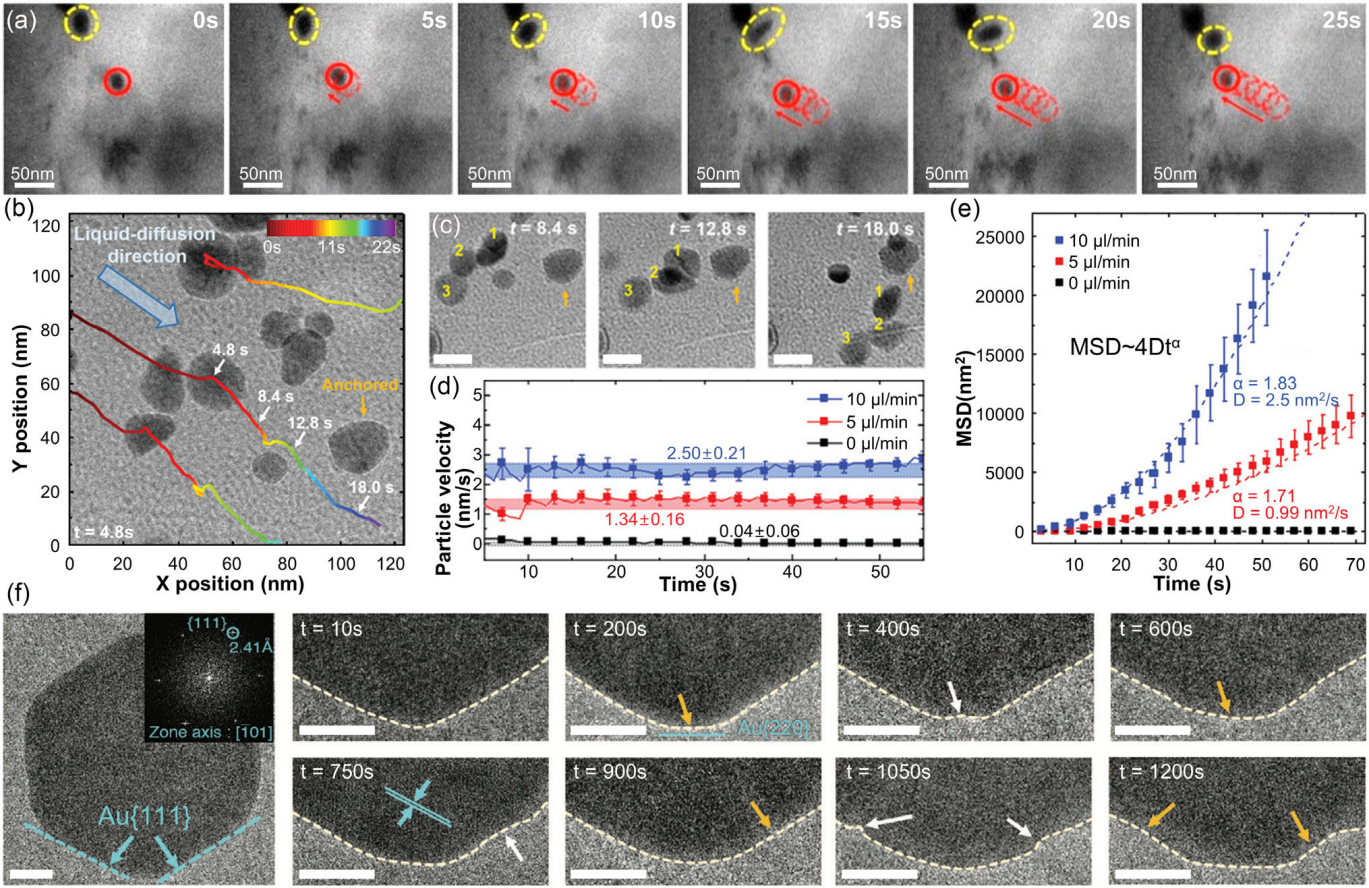
Liquid‐phase TEM studies using liquid‐flowing‐type GLCs. a) Time‐resolved TEM images showing Au nanoparticles rotating (yellow) and translating (red) within a 100 nm‐thick graphene flow cell with a Si_3_N_4_ layer. Reproduced with permission.^[^ ^191^ ^]^ Copyright 2020, American Chemical Society. b) Collective diffusion of multiple nanoparticles under continuous liquid flow. c) Time‐series TEM images showing the movement of nanoparticles. Scale bar: 20 nm. d) Nanoparticle velocity as a function of liquid flow rate. e) Mean‐square displacements of nanoparticles under different flow rates. The dotted lines represent fitted MSDs. f) Time‐series TEM images showing oxidative etching of an icosahedral Au nanoparticle under etchant flow. Scale bar: 20 nm. Reproduced with permission.^[^ ^194^ ^]^ Copyright 2021, Wiley‐VCH.

Leveraging these advancements, liquid‐flowing‐type GLCs have facilitated studies of nanoscale phenomena under precisely controlled reaction conditions. For instance, Dunn et al. investigated various dynamic behaviors of gold nanoparticles (AuNPs) dispersed in aqueous media.^[^ ^191^ ^]^ Initially, deionized water was injected through the fluidic channel with the electron beam blanked to avoid electron‐beam‐induced artifacts. Subsequently, a phosphate‐buffered solution containing AuNPs was infused into the nanofluidic channel. An observed AuNP (highlighted by the yellow dashed line) displayed shrinkage and growth in the liquid environment. Additionally, the AuNP showed rotational motion with changes in its projected area (**Figure** **9**a). Another AuNP (marked by the red line) exhibited translational movement toward a neighboring AuNP, with a translational velocity of 2.7 nm s^−1^ following its rotational motion.

Interestingly, the measured translational and rotational velocities were significantly slower than the diffusivity predicted by the Stokes–Einstein equation,^[^ ^196^, ^197^ ^]^ suggesting that electron‐beam‐induced convective forces and interparticle interactions predominantly influenced the observed motions. Furthermore, collective motions of nanoparticles were observed, including the translational movement of AuNP pairs toward each other and their subsequent correlated motion, maintaining a consistent interparticle distance. These observations illustrate that liquid‐flowing‐type GLCs can effectively resolve both individual single‐nanoparticle motions and correlated particle interactions with high spatial resolution.

In addition to the dynamic motions of nanoparticles, the crystallization and growth behavior of uranyl acetate (UA) were explored using liquid‐flowing‐type GLCs.^[^ ^191^ ^]^ A mixed solution of UA and phosphate buffer was introduced into the liquid cell, and electron beam irradiation subsequently triggered nucleation. UA crystals exhibited rotational motion and rapid dissolution—a behavior attributed to their unstable, high‐energy disordered structures.

Liquid‐flowing‐type GLCs further allowed visualization of particle motions driven by continuous solution flow within the nanofluidic channels.^[^ ^194^ ^]^ The particles exhibited collective movements correlated with the infusion rates of the solution, rather than displaying random particle motions. Increased infusion rates accelerated both the translational velocities and the diffusion coefficients of the nanoparticles (Figure 9b–e). Additionally, selective etching behavior of AuNPs was observed by introducing an etchant into the fluidic channel (Figure 9f). The etching process is preferentially initiated at the nanoparticle corners, where the energy is higher than at other sites, providing insight into the anisotropic dissolution mechanism.

Beyond inorganic materials, liquid‐flowing‐type GLCs enabled high‐contrast imaging of soft and beam‐sensitive biological specimens, including polymer beads, liposomes, and bacteria.^[^ ^194^ ^]^ The preserved imaging contrast in these soft specimens was attributed to the thin liquid layer maintained below 100 nm in thickness. Furthermore, the achievable spatial resolution using this configuration was quantitatively evaluated through analysis of the contrast transfer function (CTF), revealing an information limit of 3.7 Å—comparable to that achievable in cryo‐EM.^[^ ^198^ ^]^ This finding implies that liquid‐flowing‐type GLCs can facilitate atomic‐scale visualization of biomolecular dynamics in liquid environments while minimizing electron beam‐induced damage. Consequently, liquid‐flowing‐type GLCs present promising opportunities for high‐resolution imaging of biological specimens.

Although liquid‐flowing‐type GLCs enable the monitoring of reaction trajectories initiated through the controlled introduction of external reactants, sample drift induced by continuous liquid flow presents challenges for prolonged high‐resolution imaging. Moreover, the limited number of fluidic channels restricts statistical analysis based on repetitive experiments, in contrast with static veil‐type and well‐type GLCs, which inherently offer numerous independent observation opportunities. Additionally, the complex fabrication procedures required for liquid‐flowing‐type GLCs contribute to increased manufacturing costs, thereby limiting their extensive use. Consequently, the development of novel GLC configurations that enable on‐demand initiation of reactions combined with sustained imaging stability remains essential.

## 5 Mixing‐Type GLCs

To address the inherent limitations associated with liquid‐flowing‐type GLC TEM—such as sample drift induced by liquid flow and restricted observations due to a limited number of liquid channels—advanced GLC designs have recently been developed. Specifically, mixing‐type GLCs incorporating electron‐beam‐sensitive 2D membranes, including MoS_2_ and graphene, have been introduced, enabling both high‐resolution imaging and precisely controlled chemical mixing.^[^ ^103^, ^111^ ^]^ These novel designs synergistically integrate the advantages of veil‐type and liquid‐flowing‐type GLCs, facilitating the controlled and on‐demand introduction of chemical reactants.

Atomically thin 2D membranes, which show electron‐beam sensitivity and impermeability to liquids, are ideal materials for enabling electron‐beam‐controlled chemical mixing in liquid cells. Strategically positioning these membranes between two distinct liquid layers allows precise regulation of liquid mixing triggered by electron‐beam‐induced membrane perforation. This mechanism leverages the electron beam sensitivity of 2D membranes to initiate controlled mixing, enabling the exploration of targeted chemical reaction pathways.

**Figure 10:**
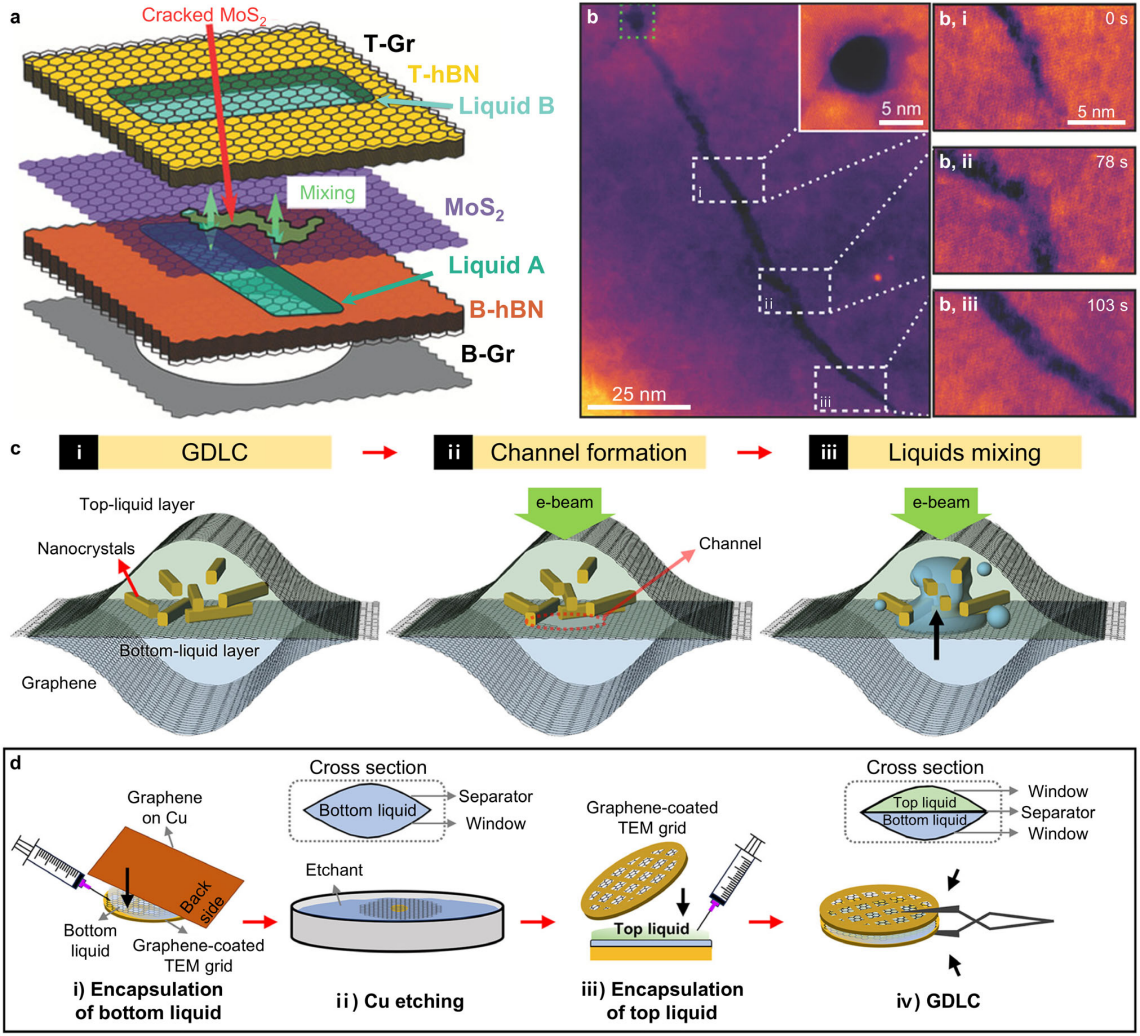
Structure and fabrication of mixing‐type GLCs. a) Schematic illustration of mixing‐type GLCs. b) Pore formation in an MoS_2_ separator induced by beam irradiation. The pore extends into a 115 nm‐long, 2–5 nm‐wide crack that allows liquid mixing. Reproduced with permission.^[^ ^111^ ^]^ Copyright 2021, Wiley‐VCH. c) Working mechanisms of the GDLC. d) Fabrication process for the GDLC. Reproduced with permission.^[^ ^103^ ^]^ Copyright 2023, American Chemical Society.

Kelly et al. initially developed mixing‐type GLCs by integrating an atomically thin MoS_2_ membrane as a separator between two discrete static liquid layers.^[^ ^111^ ^]^ Fabrication of these mixing‐type GLCs involved lithographically patterned hBN spacers, vertically stacked and tightly sealed by few‐layer graphene windows (**Figure** **10**a). A mono‐ or bilayer MoS_2_ membrane functioned as a spatial barrier separating the upper and lower liquid layers. The entire heterostructure was supported by a SiN_*x*_ support. This configuration effectively separated two independent liquid layers due to the impermeability of the MoS_2_ separator and graphene windows to liquids. The MoS_2_ separator, graphene windows, and hBN spacers were combined through van der Waals forces. Consequently, the mixing‐type GLC structure achieved precise control of liquid thickness, with the total overlapping liquid layer thickness maintained below 25 nm.

The initiation of liquid mixing within this GLC configuration was precisely controlled through the electron‐beam‐induced formation of cracks in the separator membrane. Simultaneously, the targeted chemical reactions began to be observed in real‐time. The initiation and subsequent propagation of these cracks, extending several hundred nanometers, were induced by knock‐on damage to the MoS_2_ membrane.^[^ ^128^, ^199^ ^]^ Consequently, this controlled crack formation precisely defined the specific location where chemical reactions commenced (Figure 10b). Compared to liquid‐flowing‐type GLCs, this liquid cell design offers several distinct advantages. First, initial reaction stages can be accurately captured within the selected field of view. Second, repeated observations of multiple independent reaction processes are achievable, as mixing occurs exclusively within each pair of overlapping liquid layers. Lastly, chemical reactions predominantly begin to progress along the crack trajectories, ensuring reaction propagation into the adjacent regions exposed to relatively low electron beam intensities.

Similarly, Ma et al. introduced graphene membranes as separators within mixing‐type graphene double‐liquid‐layer cells (GDLCs), which regulate the liquid mixing between two spatially separated liquid layers through graphene separator degradation.^[^ ^103^ ^]^ The structure of GDLCs maintains the basic design of veil‐type GLCs, incorporating an additional graphene membrane (typically single‐ or trilayer) as a separator. Utilizing graphene as the separator significantly reduced the background contrast compared with MoS_2_, primarily due to the lower Z‐contrast of carbon atoms relative to molybdenum. In GDLCs, mixing between liquid layers is initiated and regulated through electron beam irradiation via knock‐on damage to the graphene separator membrane and separator degradation induced by radiolysis products (Figure 10c).^[^ ^200^, ^201^ ^]^ The threshold required to induce knock‐on damage to the carbon atoms of graphene is ≈86 kV,^[^ ^200^ ^]^ which is sufficiently below the typical acceleration voltage of the electron beam in TEM ranging from 200 to 300 kV. External multilayer graphene membranes, consisting of 6–8 layers, ensure robust sealing of liquid layers from the high‐vacuum environment. In this configuration, the thinner graphene separator would preferentially degrade under electron beam irradiation, while the thicker outer membranes maintain liquid encapsulation.

The GDLC fabrication method is straightforward, adopting established direct‐transfer techniques and eliminating intricate micropatterning procedures by employing wet etching of CVD graphene substrates.^[^ ^103^, ^123^ ^]^ The fabrication steps include the following: 1) the initial transfer of multilayer graphene sheets—serving as outer imaging windows and vacuum sealing membranes—onto a holey carbon TEM grid, followed by attachment of an additional graphene sheet supported by a Cu substrate to the prepared graphene‐coated grid, thereby forming liquid pockets through van der Waals interactions between the facing graphene sheets; 2) removal of the Cu substrate by etching with an ammonium persulfate solution; 3) encapsulation of the second liquid layer by placing another graphene‐coated grid onto the prepared liquid cell; and 4) formation of the GDLC structure (Figure 10d). AFM analysis confirmed that ≈80% of the entire region formed the double‐liquid‐layer structure.

Consequently, mixing‐type GLCs are useful for investigating liquid‐phase chemical reactions with high spatial resolution and precise temporal control over reaction initiation. By spatially separating two distinct liquid layers and enabling their controlled mixing through electron‐beam‐induced perforation of an atomically thin membrane, mixing‐type GLCs significantly expanded the scope of observable nanoscale processes beyond conventional electron‐beam‐induced processes. This capability allows real‐time tracking and visualization of intricate chemical processes—including nucleation, growth, and degradation of nanomaterials—from their earliest stages under reaction conditions closely mimicking realistic chemical environments.

Kelly et al. captured the calcium carbonate (CaCO_3_) formation pathway in real‐time using mixing‐type GLCs, with distinct nucleation and growth stages fully resolved from the earliest moment of precursor mixing.^[^ ^111^ ^]^ Previous observations of CaCO_3_ nucleation were limited to electron‐beam‐induced initiation due to the structural constraints of veil‐type GLCs.^[^ ^173^ ^]^ In the mixing‐type GLC, two separate precursor solutions—containing CaCl_2_ and Na_2_CO_3_, respectively—were individually loaded into isolated chambers, remaining separated until mixing was initiated through electron‐beam‐induced damage to a MoS_2_ membrane. This enabled the direct visualization of the multistep growth process with subsecond temporal resolution.

At the initial stage of the reaction, irregularly shaped, dense liquid globules appeared near the cracks, which were attributed to spinodal decomposition and subsequent liquid–liquid phase separation.^[^ ^202^, ^203^ ^]^ These globules gradually became hydrated and transformed into small amorphous particles, evolving through distinct, multistage growth pathways. Initially, particles grew through ripening, followed by structural transformation into a sphere‐like shape. Subsequently, coalescence‐mediated growth became the dominant mechanism, reducing the total number of particles while increasing the average particle size. The nucleation and growth behavior of these nanoparticles was understood through the formation of prenucleation clusters (PNCs)^[^ ^204^ ^]^ characterized by amorphous and mobile features consistent with early‐stage, nonclassical nucleation pathways.^[^ ^153^, ^205^ ^]^ Notably, the formation and growth of PNCs extended beyond the region directly irradiated by the electron beam, indicating that mixing‐induced reactions occurred throughout the entire liquid chamber. Finally, after ≈14 min of aging under beam‐blanked conditions, the initially amorphous PNCs transformed into crystalline rhombohedral calcite nanocrystals.

**Figure 11:**
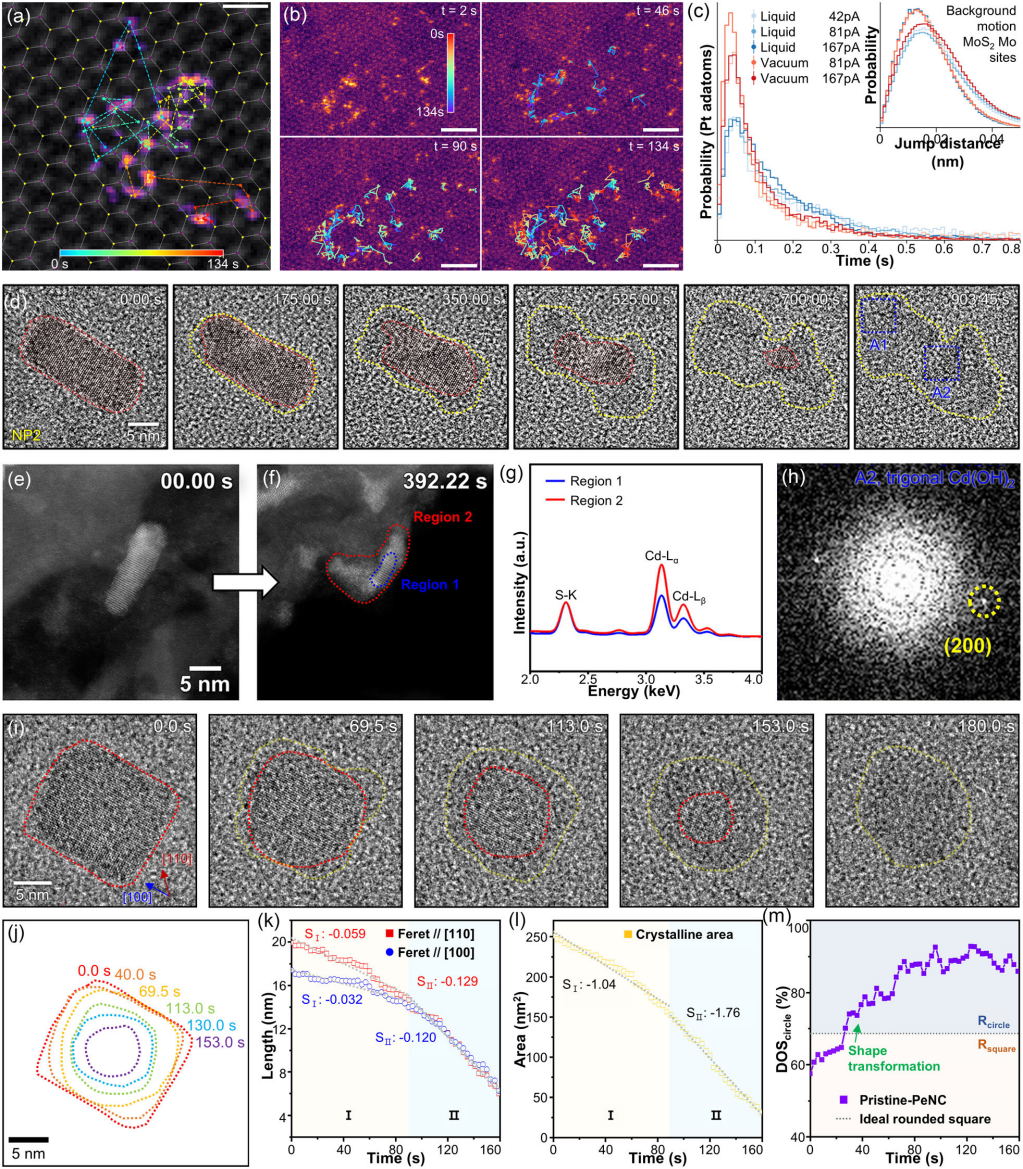
Liquid‐phase TEM studies using mixing‐type GLCs. a) Trajectory of a single Pt adatom tracked for 134 s. b) Time‐dependent trajectories of Pt atom ensembles in the liquid cell. Scale bar: 2 nm. c) Histograms of frame‐to‐frame jump lengths for Pt atoms in liquid (blue) and in vacuum (red) under three beam currents. Reproduced with permission.^[^ ^206^ ^]^ Copyright 2022, Springer Nature. d) Time‐resolved TEM images showing degradation of CdS nanorods induced by water. e,f) HAADF‐STEM images acquired before and after amorphous‐phase–mediated decomposition. g) EDS spectra from regions 1 and 2 in (f). h) FFT pattern from region A2 in d). Reproduced with permission.^[^ ^103^ ^]^ Copyright 2023, American Chemical Society. i) Time‐series TEM images showing water‐induced degradation of PeNCs. j) Changes in crystalline area during degradation. k) Changes in Feret diameter along the [110] and [100] directions as a function of time. Dashed lines indicate linear fits for stages I and II. l) Changes in projected crystalline area as a function of time with corresponding linear fits. m) Quantitative analysis of PeNC shape evolution during degradation. The dotted line corresponds to the ideal rounded square (68.6%). Reproduced with permission.^[^ ^104^ ^]^ Copyright 2025, Elsevier.

Remarkably, the introduction of atomically thin membranes (e.g., graphene and MoS_2_) and a well‐defined liquid thickness below 70 nm in the double graphene liquid cell (DGLC) enabled the tracking of single‐atom dynamics influenced by the crystalline MoS_2_ membrane in the liquid phase.^[^ ^206^ ^]^ In this setup, two distinct liquid layers—a H_2_PtCl_6_ in a water/isopropanol mixture solution and a Pt‐free water/isopropanol mixture solution—were separately encapsulated above and below the MoS_2_ separator membrane. Pt adatoms were observed to adsorb onto the MoS_2_ membrane surface. High‐resolution HAADF‐STEM directly visualized and tracked the positions of over 70 000 Pt adatoms with high temporal resolution, correlating their positions to the underlying MoS_2_ lattice for statistical analysis (**Figure** **11**a,b). Pt adatoms appeared as distinct bright spots on the hexagonal lattice of single‐layer MoS_2_ due to the strong Z‐contrast of Pt relative to the background Mo and S atoms. Under vacuum conditions, Pt adatoms preferentially occupied S lattice sites. In contrast, in a liquid environment, the Pt adatoms occupied both Mo and S sites in similar proportions, attributed to the binding of oxygen atoms at sulfur vacancy sites.^[^ ^207^ ^]^ Pt adatoms in the liquid solution exhibited larger frame‐to‐frame displacements and higher surface diffusivities—exceeding 0.25 nm^2^ s^−1^—compared to vacuum conditions, wherein diffusivities remained below 0.2 nm^2^ s^−1^. This enhanced mobility is likely attributable to solvation effects and modified charge states of Pt species. Importantly, differences in diffusivity between the liquid and vacuum conditions substantially exceeded any changes induced by electron flux, indicating that the observed diffusive motion of Pt adatoms was not driven by the electron beam (Figure 11c).

Furthermore, the precise, on‐demand mixing capability between two liquid layers in mixing‐type GLCs has significantly advanced the understanding of degradation mechanisms of quantum‐sized semiconductor nanocrystals. Quantum‐sized semiconductor nanocrystals exhibit outstanding optical and electronic properties suitable for optoelectronic devices^[^ ^208^, ^209^, ^210^, ^211^, ^212^, ^213^, ^214^, ^215^ ^]^ and catalysts.^[^ ^216^, ^217^, ^218^, ^219^ ^]^ However, their poor stability has limited their commercial applications.^[^ ^220^, ^221^, ^222^ ^]^ Therefore, understanding the degradation pathways of quantum‐sized semiconductor nanocrystals is essential for the development of synthetic strategies to improve their optical and structural stability.^[^ ^223^, ^224^, ^225^ ^]^ However, real‐time visualization of these complex processes has been challenging due to structural limitations inherent in conventional veil‐type GLCs. By using mixing‐type GLCs, recent studies successfully revealed the degradation mechanism of CdS nanocrystals—a representative metal‐chalcogenide semiconductor nanocrystal—by directly tracking structural transformations during water exposure in real‐time.^[^ ^103^ ^]^ These investigations showed the formation of amorphous reaction intermediates near crystalline regions, exhibiting highly dynamic characteristics (Figure 11d). The observed amorphous intermediates were identified as key components in the degradation process, significantly influencing subsequent reaction pathways. Specifically, CdS nanocrystals exhibited morphological transformations distinct from typical etching mechanisms revealed for metal nanocrystals characterized by smooth crystalline surfaces.^[^ ^96^, ^98^ ^]^ After the initial formation of concave features, etching preferentially proceeded along high‐energy crystallographic facets, specifically along the (0001) direction aligned with the c‐axis. This unique etching pattern produced rough, irregular boundaries rather than smooth crystal surfaces, likely due to the presence of amorphous intermediates that inhibited the direct exposure of pristine crystal facets.

Energy‐dispersive X‐ray spectroscopy (EDS) analyses (Figure 11e–g), complemented by fast Fourier transform (FFT) analyses of partially crystallized species (Figure 11h), conclusively identified the amorphous intermediates as Cd(OH)_*x*_ species. These intermediates are formed through reactions between CdS nanocrystals and water molecules. Consistent observations across multiple independent liquid pockets verified that the formation of these amorphous intermediates constitutes highly reproducible and characteristic degradation pathways for CdS nanocrystals. Moreover, ex situ characterization of CdS nanocrystals exposed to water further supported the generality of the observed degradation pathway.

Furthermore, the degradation trajectories of perovskite nanocrystals (PeNCs)—known for their sensitivity to the electron beam^[^ ^225^ ^]^—were visualized at atomic resolution using GDLCs (Figure 11i).^[^ ^104^ ^]^ The use of graphene membranes for encapsulation, along with the spatial separation between the PeNC solution and water, enabled the direct observation of the water‐induced degradation process from the initial stages. Degradation of PeNCs proceeded through solvation of constituent ions by water molecules, where the dissolution trajectories were governed by the polarity of the exposed crystal facets. Higher surface polarity facilitated stronger interactions with water molecules, thereby significantly accelerating crystal dissolution. Rapid dissolution preferentially occurred along the [110] and [111] directions, which have higher surface polarity,^[^ ^226^ ^]^ inducing the shape transformation from nanocubes to nanospheres and revealing anisotropic dissolution behavior (Figure 11j–m). Following initial shape transformation, isotropic dissolution occurred, exhibiting comparable dissolution rates irrespective of crystallographic direction. Notably, surface ligands detached from crystal surfaces during dissolution, indicating that surface ligands likely significantly influence nanocrystal stability upon water exposure.

Notably, the introduction of halide‐ion‐pair ligands—didodecyldimethylammonium bromide (DDAB)^[^ ^227^ ^]^—suppressed the initial anisotropic dissolution and enhanced both optical and structural stability of PeNCs upon water exposure. The higher binding energy of DDAB,^[^ ^228^ ^]^ compared to the original surface ligands (i.e., oleic acid and oleylamine), delayed the exposure of polar {110} and {111} facets and the formation of surface defects that facilitate crystal dissolution.^[^ ^229^ ^]^ Furthermore, the passivation of PeNCs with hydrophobic polymer ligands such as poly(methyl methacrylate*‐*co‐methacrylic acid) (P(MMA*‐*co‐MAA)) also improved stability, providing insight into tailoring nanocrystal surface chemistry for enhanced durability in practical applications.

Mixing‐type graphene GLCs broaden the scope of observable nanoscale processes by incorporating a liquid‐separator membrane that can be selectively perforated to initiate controlled, mixing‐induced reactions. This design allows the encapsulation and regulated mixing of two distinct liquid solutions within a single cell, thus closely replicating reaction conditions that typically occur outside the vacuum environment of TEM columns. Consequently, detailed insights into the structural evolution of nanomaterials during various reactions become accessible.

## 6 Liquid Cells Based on Alternative Membrane Materials Beyond Graphene

Although GLCs using atomically thin graphene membranes are extensively utilized across various liquid cell designs due to their intrinsic advantages, alternative window membranes have also been actively explored for characterizing diverse liquid‐phase reactions and analyzing the structure of nanomaterials in liquid environments.

**Figure 12:**
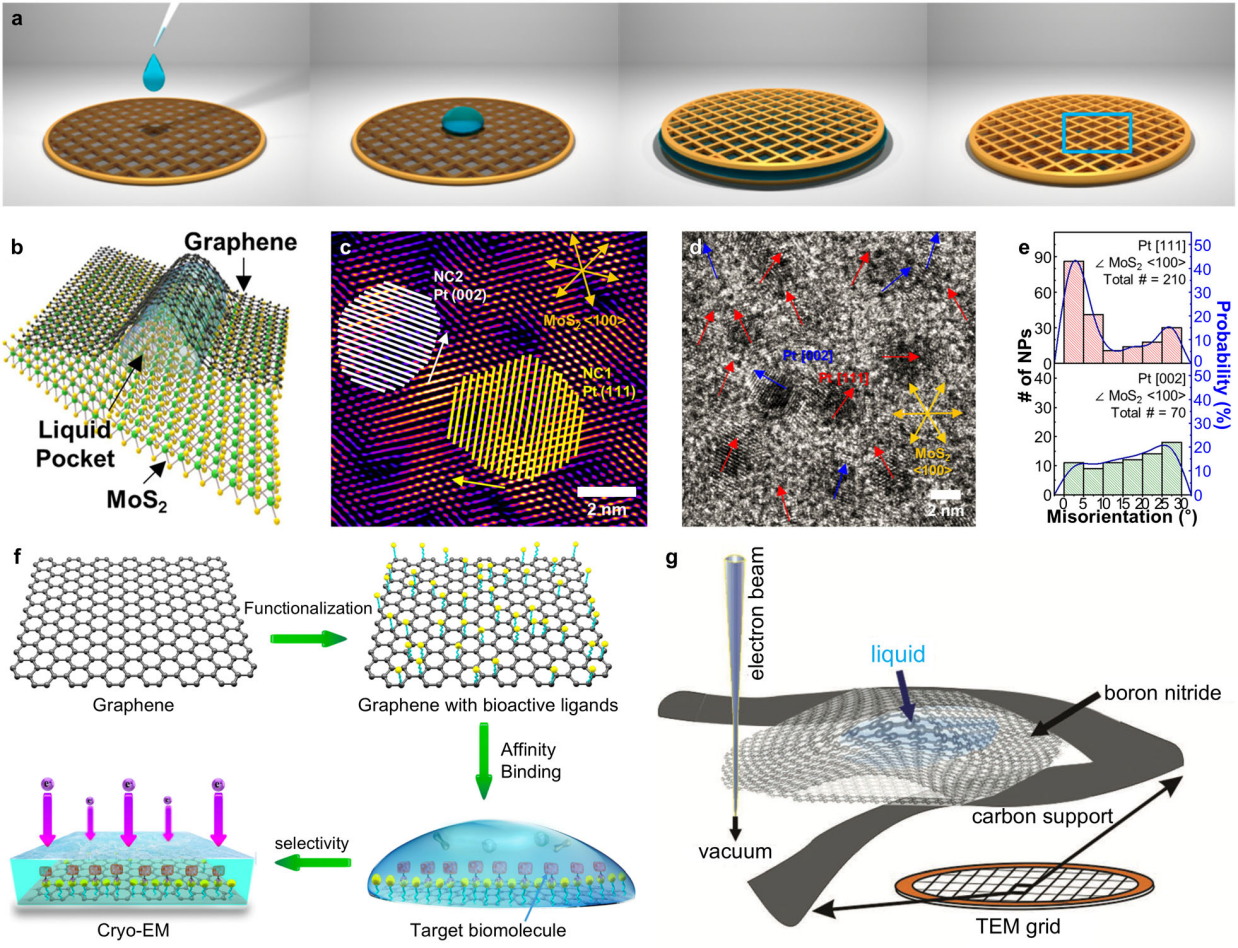
Liquid cells utilizing alternative membrane materials beyond graphene. a) Fabrication process for carbon film liquid cells. Reproduced with permission.^[^ ^230^ ^]^ Copyright 2018, Springer Nature. b) Schematic illustration showing the structure of MoS_2_ liquid cells. c) Overlay of Pt lattice fringes on the inverse‐FFT image of the MoS_2_ membrane. d) TEM image of as‐grown Pt nanocrystals. Blue and red arrows indicate the Pt [111] and [002] directions, respectively. e) Histograms of misorientation angles between Pt [111], [002], and the MoS_2_ ⟨100⟩ direction. Reproduced with permission.^[^ ^243^ ^]^ Copyright 2019, American Chemical Society. f) Schematic of graphene membranes functionalized with bioactive ligands that selectively bind target proteins for cryo‐EM. Reproduced with permission.^[^ ^245^ ^]^ Copyright 2019, American Chemical Society. g) Aloof‐mode EELS measurements with boron nitride liquid cells. Reproduced with permission.^[^ ^249^ ^]^ Copyright 2018, Wiley‐VCH.

Liquid cells using amorphous carbon films as encapsulating membranes have been extensively utilized due to their simple fabrication processes, significantly enhancing experimental reproducibility. Unlike GLCs, which involve relatively complex fabrication processes requiring precise alignment, delicate transfer steps, and careful handling to avoid membrane rupture, carbon film liquid cells offer significantly simplified fabrication procedures. Carbon film liquid cells are typically fabricated using commercially available TEM grids with metal meshes. A small droplet of the liquid sample is directly deposited between two TEM grids, each with carbon‐coated surfaces facing inward without complex membrane transfer steps. Excess liquid is subsequently evaporated under ambient conditions, forming stable liquid pockets between the carbon films (**Figure** **12**a).^[^ ^230^ ^]^ The thickness of commercially available carbon films on TEM grids generally ranges from a few to several tens of nanometers, facilitating high‐resolution TEM imaging with high contrast. Due to their simplified fabrication process, carbon film‐based liquid cells have been widely utilized to study various dynamic processes, including nanoparticle growth,^[^ ^230^ ^]^ solid–liquid‐gas interfacial reactions,^[^ ^231^, ^232^, ^233^ ^]^ etching,^[^ ^233^, ^234^, ^235^ ^]^ and deformations.^[^ ^236^ ^]^

For instance, carbon film liquid cells are used to capture atomic‐resolution, time‐series TEM images of ligand‐mediated oriented attachment between gold nanocrystals.^[^ ^202^ ^]^ Peng et al. observed two individual particles exhibiting random rotational motions approaching each other and preferentially attaching along their {111} facets after alignment via rotation. This facet‐specific attachment was promoted by the relatively weaker ligand binding energies at {111} facets compared to {100} facets. Carbon film liquid cells have also been used to study gold nanoparticle etching at the solid–liquid–gas interface in hydrobromic acid solutions. Wang et al. revealed an enhanced local reaction rate driven by the proximity of oxygen nanobubbles located within ≈1 nm of nanoparticle surfaces.^[^ ^233^ ^]^ This increased local reaction rate was attributed to the adsorption of oxygen nanobubbles onto nanoparticle surfaces via van der Waals forces, facilitating direct oxygen transport. This finding highlights the complexity of reaction mechanisms introduced by multiphase interactions. Additionally, carbon film liquid cells enabled the investigation of deformation behaviors of semiconductor nanocrystals during superlattice transitions. Wang et al. captured anisotropic elongation of PbSe nanocrystals along internanocrystal axes, accompanied by simultaneous contraction in the perpendicular directions.^[^ ^236^ ^]^ Interestingly, these deformation behaviors were reversible in the absence of attachment, whereas attachment led to permanent shape transformations.

In addition to amorphous carbon films, ultrathin SiN_*x*_ with thicknesses around 10 nm are considered promising alternatives, exhibiting significantly improved electron transparency and reduced electron scattering compared to conventional SiN_*x*_ membranes in microfabricated liquid cells with thicknesses of several tens of nanometers.^[^ ^237^, ^238^ ^]^ These ultrathin SiN_*x*_ membranes facilitated enhanced sensitivity and resolution in high‐resolution TEM imaging, electron energy loss spectroscopy (EELS), and EDS analyses. Koo et al. introduced ultrathin (≈10 nm) SiN_*x*_ membranes supported by heavily doped substrates, thus enhancing mechanical robustness, electron dose tolerance, and the information limit.^[^ ^237^ ^]^ This design significantly improved the signal‐to‐noise ratio in EELS and EDS analyses by reducing electron scattering and background signals. For instance, the EELS spectra of PdH_*x*_ exhibited clearly distinguishable peaks corresponding to *β*‐PdH_*x*_ and H_2_ gas, indicating the phase transformation through hydrogen absorption. Additionally, the EDS spectra collected from SiN_*x*_ cells filled with Ar gas showed more than 14‐fold enhancement in the Ar/Si signal ratio compared to cells fabricated with thicker (50 nm) SiN_*x*_ membranes.

Additionally, Liao et al. utilized liquid cells with 10 nm‐thick SiN_*x*_ membrane windows to visualize the growth process of Pt nanocrystals.^[^ ^238^ ^]^ Facet‐selective growth processes were captured, wherein the {110} and {111} crystal facets continued growing even after growth on {100} facets stopped, thereby facilitating the formation of nanocubes. Time‐series TEM imaging revealed atomic‐level attachment processes onto crystal facets, confirming a layer‐by‐layer growth mechanism. Additionally, fluctuations in growth rates during the transformation into nanocubes were observed, reflecting local variations in the reaction environment throughout the nanocrystal growth process.

Beyond chemically inert membranes such as graphene, amorphous carbon films, and SiN_*x*_,^[^ ^239^, ^240^, ^241^, ^242^ ^]^ membranes with distinctive physicochemical properties have been actively explored, leveraging their unique characteristics to investigate interfacial interactions between nanomaterials and membrane surfaces and facilitate advanced spectroscopic analyses.

2D MoS_2_ membranes have enabled the investigation of interfacial interactions between the membrane and nanocrystals during the growth process.^[^ ^243^ ^]^ Yang et al. introduced MoS_2_ membranes into liquid cells using a polymer‐free transfer method, in which the underlying SiO_2_ layer was selectively etched using hydrofluoric acid to minimize mechanical damage to the MoS_2_ membranes. The liquid pockets were subsequently formed by encapsulating liquids between graphene and MoS_2_ membranes (Figure 12b), yielding anisotropic, pseudo‐rectangular liquid pockets, distinct from the irregularly shaped pockets typically observed in conventional GLCs. Notably, MoS_2_ membranes actively interacted with deposited nanomaterials, in contrast to graphene, which is chemically inert. Observations of Pt nanocrystal growth trajectories revealed van der Waals epitaxial relationships with the MoS_2_ substrate. Specifically, Pt nanocrystals preferentially aligned their crystallographic ⟨111⟩ orientations along the ⟨100⟩ direction of the underlying MoS_2_ lattice (Figure 12c–e). Such van der Waals epitaxial growth may stabilize the Pt–MoS_2_ heterostructures, facilitating controlled nanoparticle growth.^[^ ^244^ ^]^ Consequently, the function of the MoS_2_ membrane extends beyond mere mechanical liquid encapsulation, providing additional opportunities for the controlled assembly of nanostructures and manipulation of interfacial chemistry within liquid‐phase reaction environments.

Furthermore, chemically functionalized 2D membranes have been explored extensively for single‐particle cryo‐EM. Although pristine graphene lacks chemical specificity, posing challenges in orienting biomolecules during cryo‐EM sample preparation, chemical functionalization of graphene surfaces has effectively addressed these limitations. Functionalization of graphene surfaces with bioactive ligands enabled the selective targeting of biomolecules.^[^ ^245^ ^]^ Liu et al. successfully functionalized graphene membranes with nickel‐nitrilotriacetic acid (Ni‐NTA), specifically targeting His‐tagged proteins (Figure 12f). The covalent coordination of Ni‐NTA to graphene facilitated precise and stable binding of His‐tagged polynucleotide phosphorylase (PNPase) and 20S proteasomes. This approach significantly reduced undesirable protein adsorption at the air–water interface—a crucial improvement for accurate 3D reconstructions in cryo‐EM.^[^ ^246^ ^]^ Additionally, the negligible background noise from the graphene substrate, along with uniformly oriented and stabilized proteins, enabled a high resolution of 3.8 Å in 3D reconstructions of 20S proteasomes.

In addition, GO functionalized with single‐stranded DNA or a thiol‐poly(acrylic acid*‐*co‐styrene) copolymer showed enhanced nonspecific electrostatic interactions with protein–nucleic acid complexes.^[^ ^247^ ^]^ This functionalization effectively prevented protein denaturation typically induced by adsorption at the air–water interface, enabling high‐resolution (≈2.3 Å) 3D reconstructions without the necessity of chemical crosslinking. Notably, this approach allowed the capture of multiple conformational intermediates of the sucrose nonfermenting 2 homolog (SNF2h)‐nucleosome complex, highlighting the significant advances achievable through targeted functionalization of graphene membranes in cryo‐EM. Collectively, these functionalized membranes reveal the potential for liquid cell TEM, enabling high‐resolution structural analysis and tracking of reaction trajectories for biomolecules within their native aqueous environments.

Additionally, hBN membranes have also emerged as an alternative 2D membrane for liquid‐phase EELS due to their superior electrical insulation and low background signals in the low‐loss energy region due to their wide bandgap.^[^ ^248^ ^]^ This property addresses limitations of GLCs, where intrinsic electrical conductivity and characteristic phonon modes of graphene membranes generate significant background signals, particularly in vibrational EELS. Vibrational EELS is a monochromated STEM‐EELS technique that probes lattice and molecular vibrations by detecting low‐energy (<1 eV) phonon excitations, enabling nanoscale mapping of vibrational properties in materials.^[^ ^249^, ^250^, ^251^ ^]^ Jokisaari et al. developed boron nitride liquid cells for high‐resolution EELS analysis,^[^ ^249^ ^]^ successfully encapsulating liquid samples between two hBN membranes (Figure 12g). This configuration enhanced EELS signal detection via an aloof beam interaction approach,^[^ ^251^ ^]^ wherein electron beam interactions occur indirectly through evanescent fields, avoiding direct beam–sample contact. Using boron nitride liquid cells, vibrational modes of water molecules, such as the O—H and O—D stretching vibrations from H_2_O and D_2_O, respectively, were clearly detected with nanometer‐scale spatial resolution. Additionally, an ≈20 meV shift in the O—H stretching vibration peak was observed, attributed to local confinement effects within the liquid pockets and interfacial interactions between water molecules and the hBN membrane surface.

Overall, advancements in window materials and cell designs continuously extend the scope and enhance the accuracy of various analyses. Therefore, the continued development of window membranes is anticipated to offer unprecedented insights into interfacial chemistry and nanoscale processes of both inorganic and biomaterials.

## 7 Conclusions and Outlook

GLCs have significantly advanced liquid‐phase TEM by enabling real‐time visualization of nanoscale dynamic processes with atomic‐level spatial resolution. Diverse GLC configurations—including veil‐type, well‐type, liquid‐flowing‐type, and mixing‐type designs—have been developed, each tailored to specific experimental challenges (**Table** **1**). Veil‐type GLCs, fabricated by simply trapping a liquid droplet between two graphene sheets, have facilitated high‐resolution visualization of liquid‐phase processes. Well‐type GLCs, characterized by precisely patterned hole arrays for liquid encapsulation, offer uniform and reproducible experimental conditions, thereby enabling effective quantitative analysis. Liquid‐flowing‐type GLCs extend the experimental scope by integrating nanofluidic channels, enabling precise modulation of chemical environments within the liquid chamber. Mixing‐type GLCs incorporate electron‐beam‐sensitive membranes separating two isolated liquid layers, allowing controlled, on‐demand mixing triggered by electron beam irradiation. This capability for precise initiation of reactions expands the range of observable nanoscale processes.

**Table 1:** Comparative summary of the key capabilities of the four representative GLC configurations.

	Veil‐type	Well‐type	Liquid‐flowing‐type	Mixing‐type
Spatial resolution	High (atomic scale)	High (atomic scale)	High (atomic scale, limited by liquid flow)	High (atomic scale)
Pocket geometry control	Limited	Allowed	Allowed	Allowed
Fabrication complexity	Low	Moderate–high	High	Variable (design‐dependent)
Reaction initiation control	Controlled by radiolysis products or pre‐mixed before loading	Controlled by radiolysis products or pre‐mixed before loading	Controlled by liquid flow	Controlled by on‐demand liquid separator perforation
Number of observations	Multiple	Multiple	Limited	Multiple

Despite significant progress, fundamental challenges related to the restricted environment intrinsic to TEM necessitate further careful consideration and the development of advanced approaches for in situ TEM studies. First, reactive radicals are continuously generated under electron beam irradiation. Although graphene can mitigate radical‐induced damage by scavenging these species, radical formation cannot be completely suppressed. Such highly reactive radicals significantly alter local chemical compositions, initiate undesired side reactions,^[^ ^252^ ^]^ and considerably influence the chemical environment within liquid cells.^[^ ^253^, ^254^, ^255^ ^]^ They also contribute to the degradation of graphene or GO windows in liquid cells,^[^ ^201^, ^256^ ^]^ thereby necessitating careful liquid cell design and fabrication to ensure the integrity of the liquid environment. Importantly, the concentration of these electron‐beam‐induced products highly depends on electron dose,^[^ ^257^ ^]^ necessitating careful evaluation of dose effects in in situ TEM imaging. Second, electron beam irradiation also induces the formation of gas bubbles, potentially altering local reaction kinetics,^[^ ^233^, ^258^ ^]^ thereby complicating the interpretation of observed processes. The dynamic behaviors of gas bubbles within GLCs^[^ ^259^ ^]^ may disrupt tracking of targeted nanoparticles, limiting prolonged imaging with high spatial resolution. Third, confined geometry of GLCs inherently restricts the volume of encapsulated liquid, constraining reactions to occur within highly restricted spatial domains. Consequently, discrepancies often emerge due to these inherent, nonequilibrium conditions, possibly differing from typical reaction environments. This complicates the validation of reaction mechanisms derived from TEM observations. Additionally, precise control of experimental parameters within GLCs—such as temperature and electrochemical potential—remains limited, thus inhibiting systematic studies of nanoscale dynamics under varying conditions.

Therefore, various complementary strategies have been actively explored to address these challenges. The introduction of chemical scavengers into the liquid medium^[^ ^253^, ^254^, ^255^, ^260^ ^]^ has proven to be an effective solution for mitigating radical effects. In particular, aliphatic alcohols, halide ions, and halide‐containing organic molecules preferentially scavenge reactive radical species.^[^ ^253^, ^261^, ^262^, ^263^, ^264^ ^]^ Substituting regular water (H_2_O) with deuterated water (D_2_O) can suppress gas bubble formation, thereby substantially extending the duration of bubble‐free imaging.^[^ ^265^, ^266^ ^]^ Furthermore, surface treatments of graphene windows to improve liquid wettability can enhance the stability of gas–liquid interfaces.^[^ ^259^ ^]^ In parallel, low‐dose imaging techniques, combined with advances in electron detector performance and computational denoising algorithms, offer comprehensive approaches for addressing the effect of radicals, gas bubbles, and dose effects, thereby enhancing the reliability of TEM observations.^[^ ^260^, ^267^, ^268^, ^269^, ^270^, ^271^ ^]^ Moreover, complementary ex situ characterization performed outside the TEM environment, along with theoretical simulations such as MD and density functional theory (DFT) calculations, provides valuable insight for validating TEM observations. Statistical analyses based on repeated observations may further strengthen the generality and reproducibility of observed processes within confined liquid environments.

Considering the progress in GLC TEM discussed in this review, future developments are expected to integrate additional functionalities—such as thermal control,^[^ ^35^, ^36^, ^37^, ^38^, ^39^, ^272^, ^273^ ^]^ electrochemical control,^[^ ^80^, ^81^, ^82^, ^83^, ^84^, ^85^, ^86^, ^87^, ^274^, ^275^, ^276^, ^277^, ^278^ ^]^ and other reaction control capabilities, including the introduction of gaseous species^[^ ^279^, ^280^, ^281^, ^282^, ^283^, ^284^, ^285^, ^286^, ^287^, ^288^ ^]^—into GLCs. Such multifunctional designs will help overcome the limitations of current cells and further expand their applicability. Continuous developments in liquid cell TEM technologies will facilitate the exploration of nanoscale processes under conditions more closely resembling real‐world environments. These advancements will broaden the scope and enhance the generality of liquid cell TEM observations, thereby narrowing the gap between fundamental insights gained through high‐resolution TEM observations and practical applications.

## Conflict of Interest

The authors declare no conflict of interest.
